# Supplementary figures and images for: Conserved but mechanistically diverse piRNA defence against endogenous retroviruses in insects
Source: EMBO Rep. 2026 Apr 2;27(9):2243–73. doi: 10.1038/s44319-026-00741-4 (PMC13172572; doi:10.1038/s44319-026-00741-4)

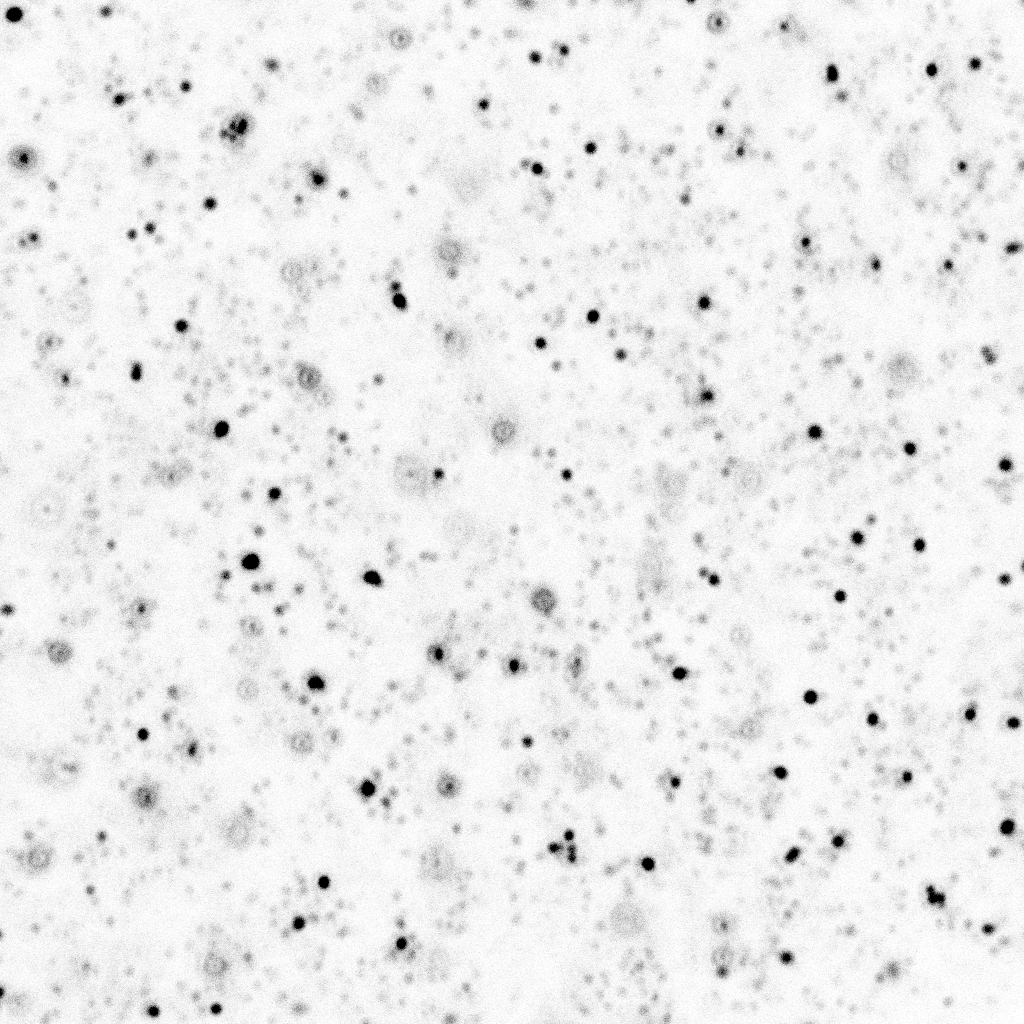

Supplement: Supplementary file 4 — Source data Fig. 2 [file 44319_2026_741_MOESM4_ESM.zip › Figure_2/2H/Anopheles_stephensi_cluster_RNA-FISH_birds-eye_FISH-only.tif]

Figure 2H

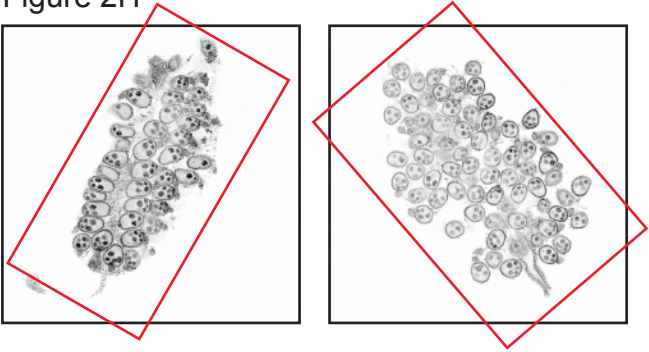

Supplement: Supplementary file 4 — Source data Fig. 2 [file 44319_2026_741_MOESM4_ESM.zip › Figure_2/2H/microscopy-images_cropping-information_Fig2H.pdf]

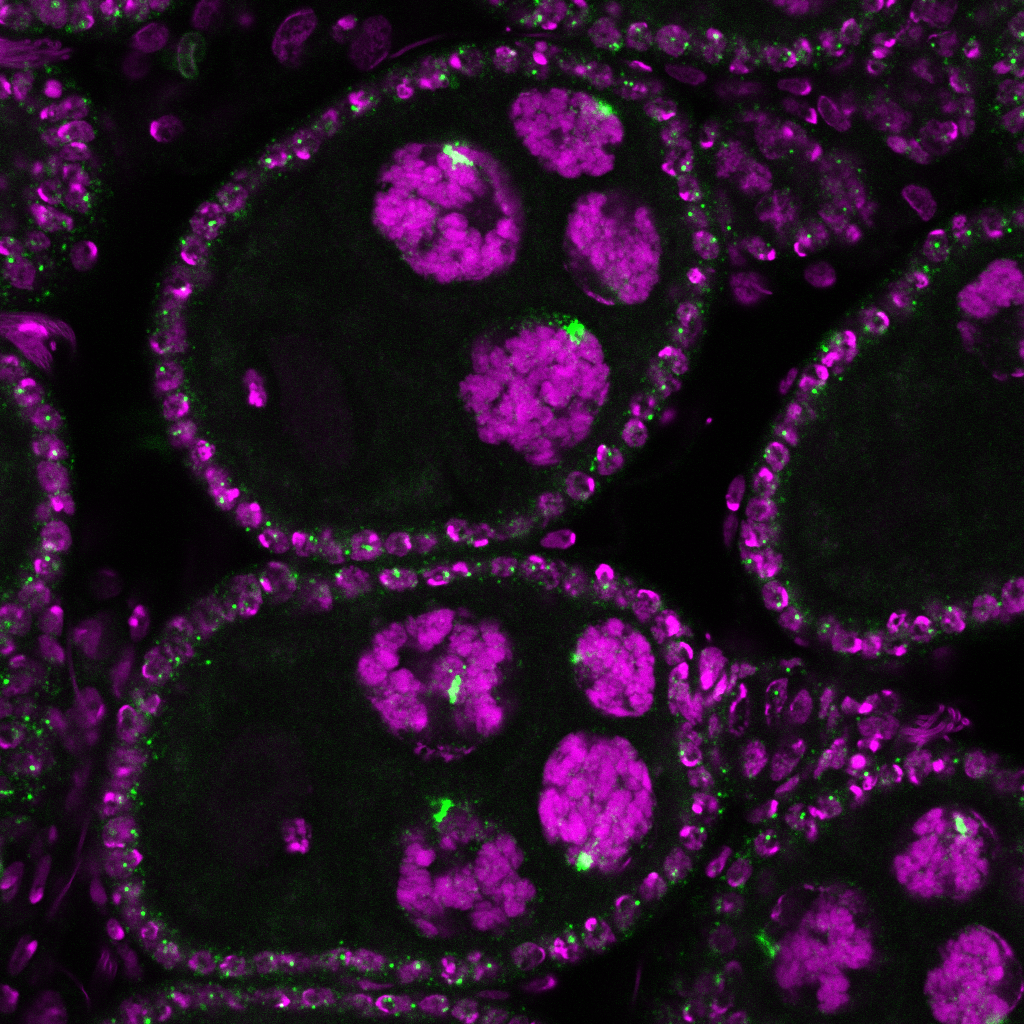

Supplement: Supplementary file 4 — Source data Fig. 2 [file 44319_2026_741_MOESM4_ESM.zip › Figure_2/2H/Anopheles_stephensi_cluster_RNA-FISH_FISH-DAPI.tif]

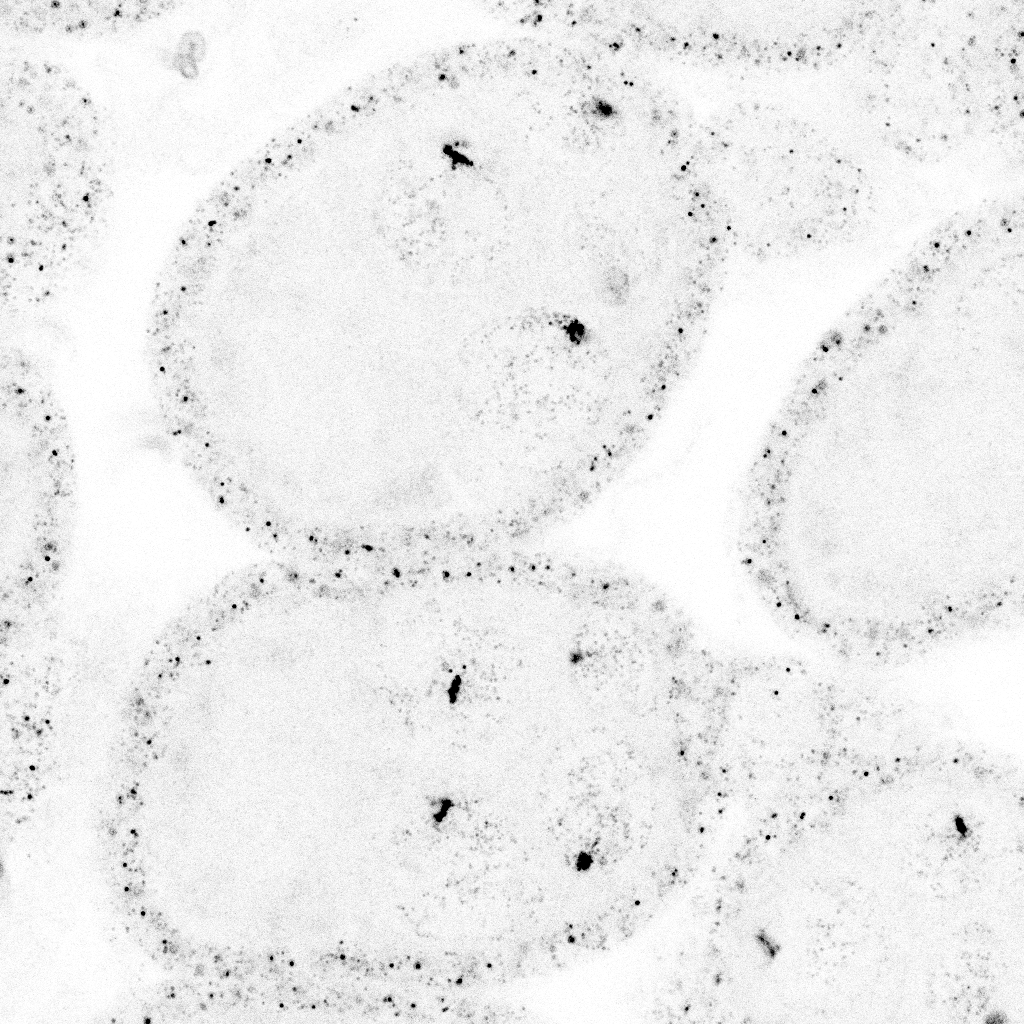

Supplement: Supplementary file 4 — Source data Fig. 2 [file 44319_2026_741_MOESM4_ESM.zip › Figure_2/2H/Anopheles_stephensi_cluster_RNA-FISH_FISH-only.tif]

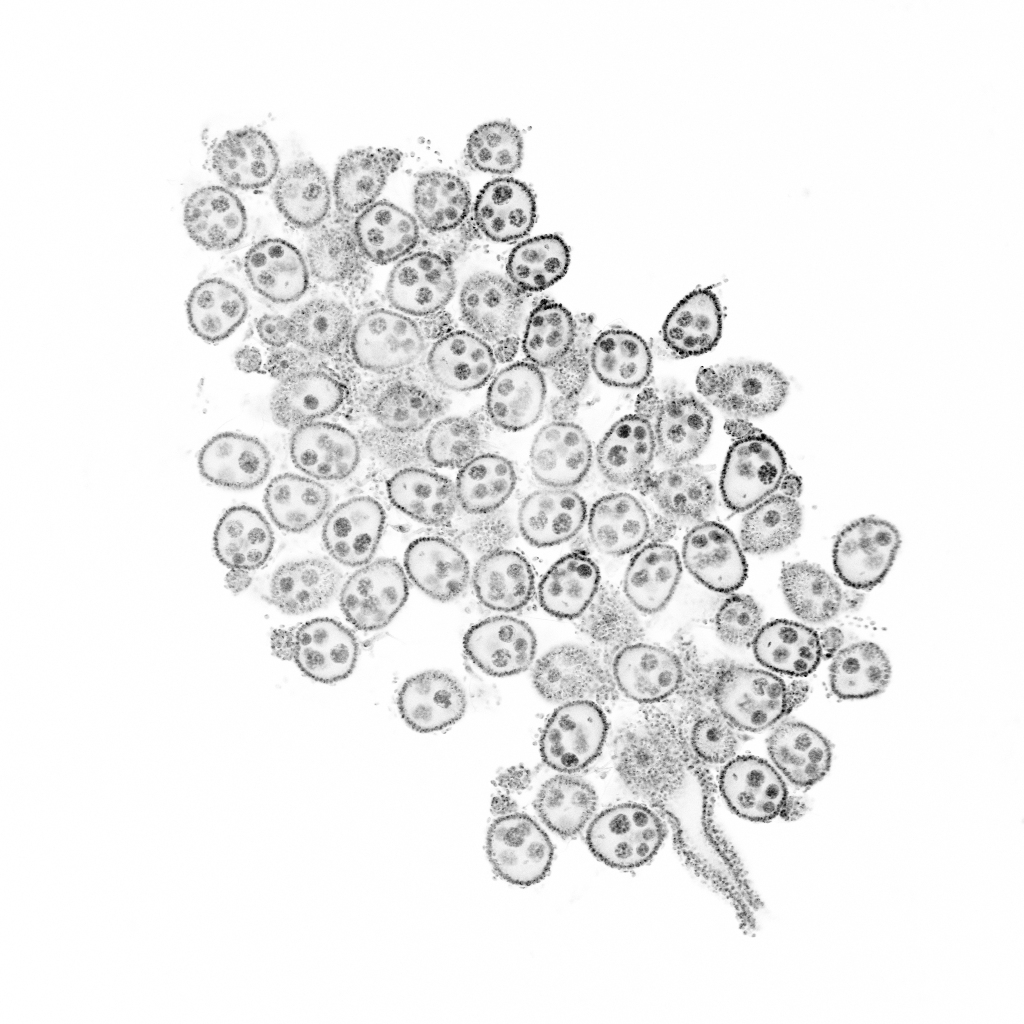

Supplement: Supplementary file 4 — Source data Fig. 2 [file 44319_2026_741_MOESM4_ESM.zip › Figure_2/2H/Anopheles_stephensi_whole-ovary_DAPI_2.tif]

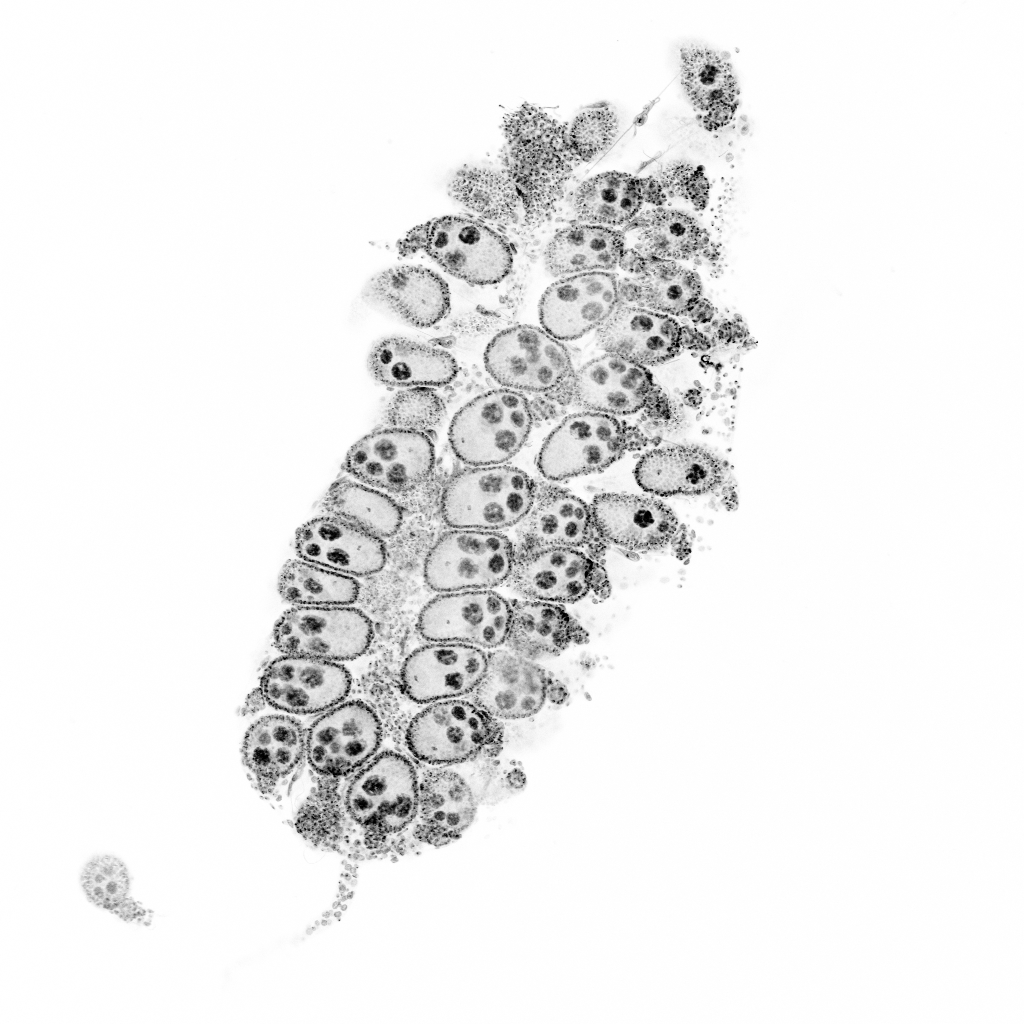

Supplement: Supplementary file 4 — Source data Fig. 2 [file 44319_2026_741_MOESM4_ESM.zip › Figure_2/2H/Anopheles_stephensi_whole-ovary_DAPI_1.tif]

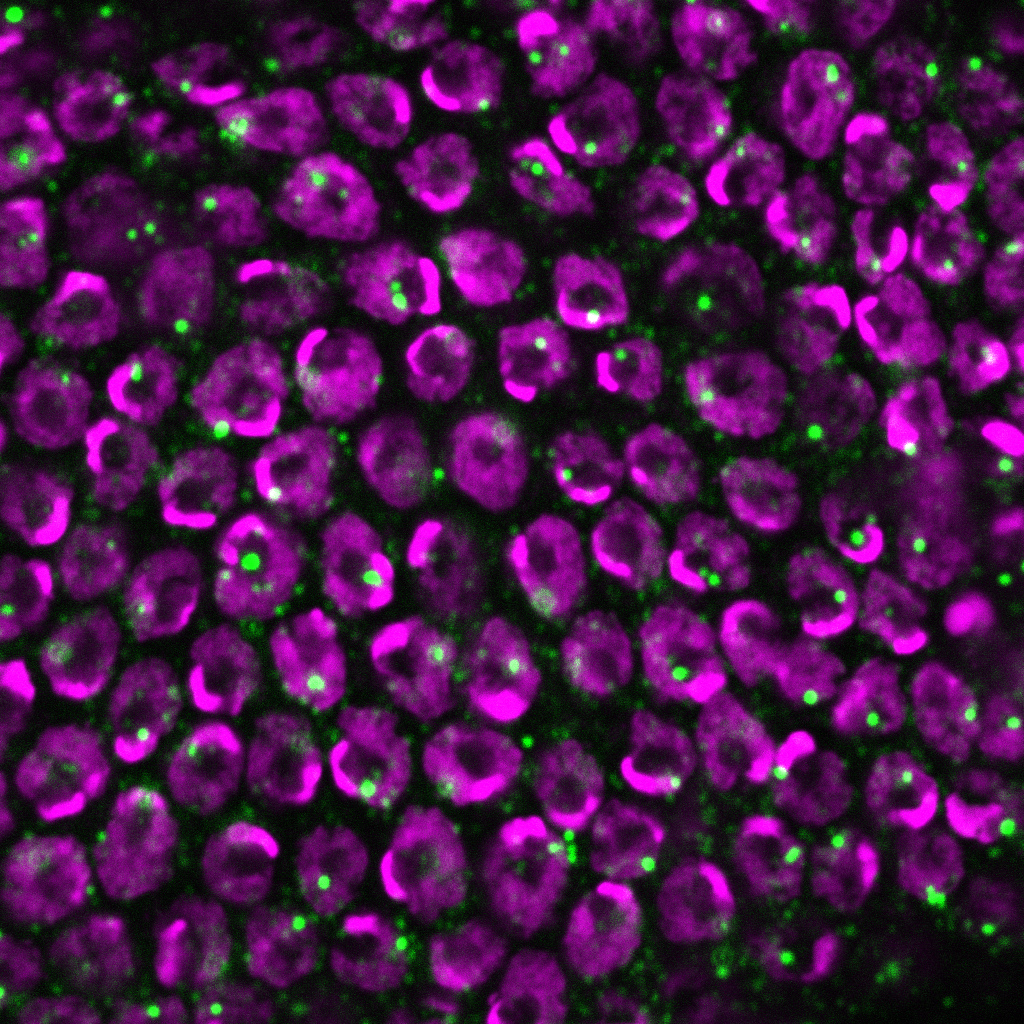

Supplement: Supplementary file 4 — Source data Fig. 2 [file 44319_2026_741_MOESM4_ESM.zip › Figure_2/2H/Anopheles_stephensi_cluster_RNA-FISH_birds-eye_FISH-DAPI.tif]

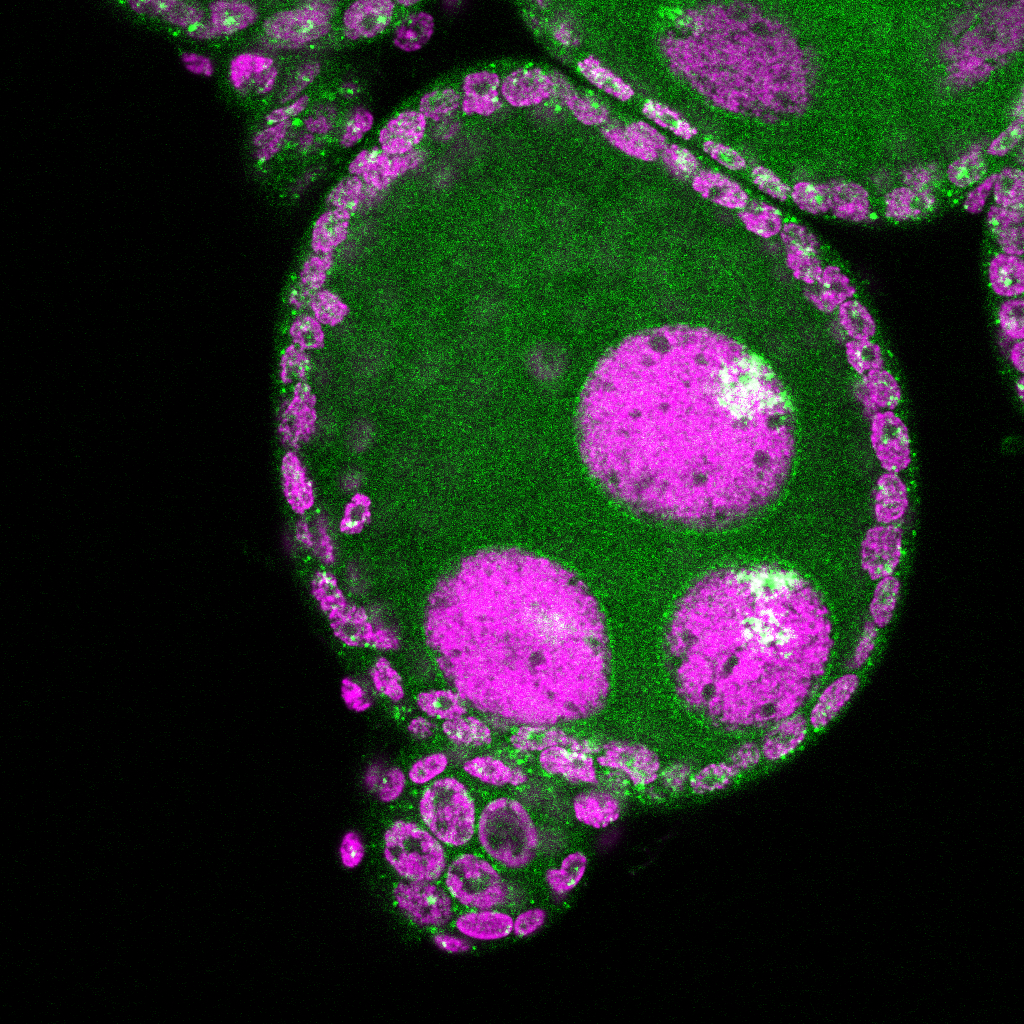

Supplement: Supplementary file 4 — Source data Fig. 2 [file 44319_2026_741_MOESM4_ESM.zip › Figure_2/2C/Aedes_aegypti_cluster3_RNA-FISH_FISH-DAPI.tif]

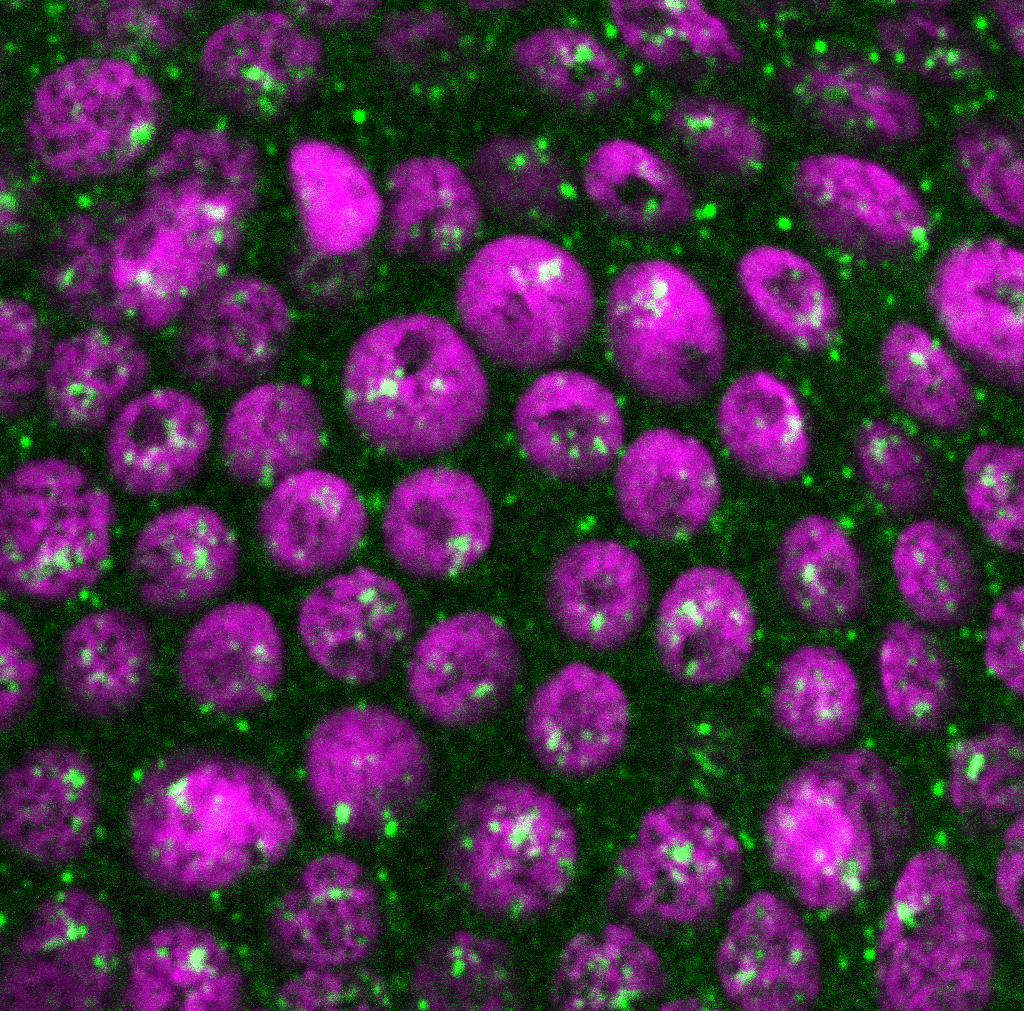

Supplement: Supplementary file 4 — Source data Fig. 2 [file 44319_2026_741_MOESM4_ESM.zip › Figure_2/2C/Aedes_aegypti_cluster3_RNA-FISH_birds-eye_FISH-DAPI.tif]

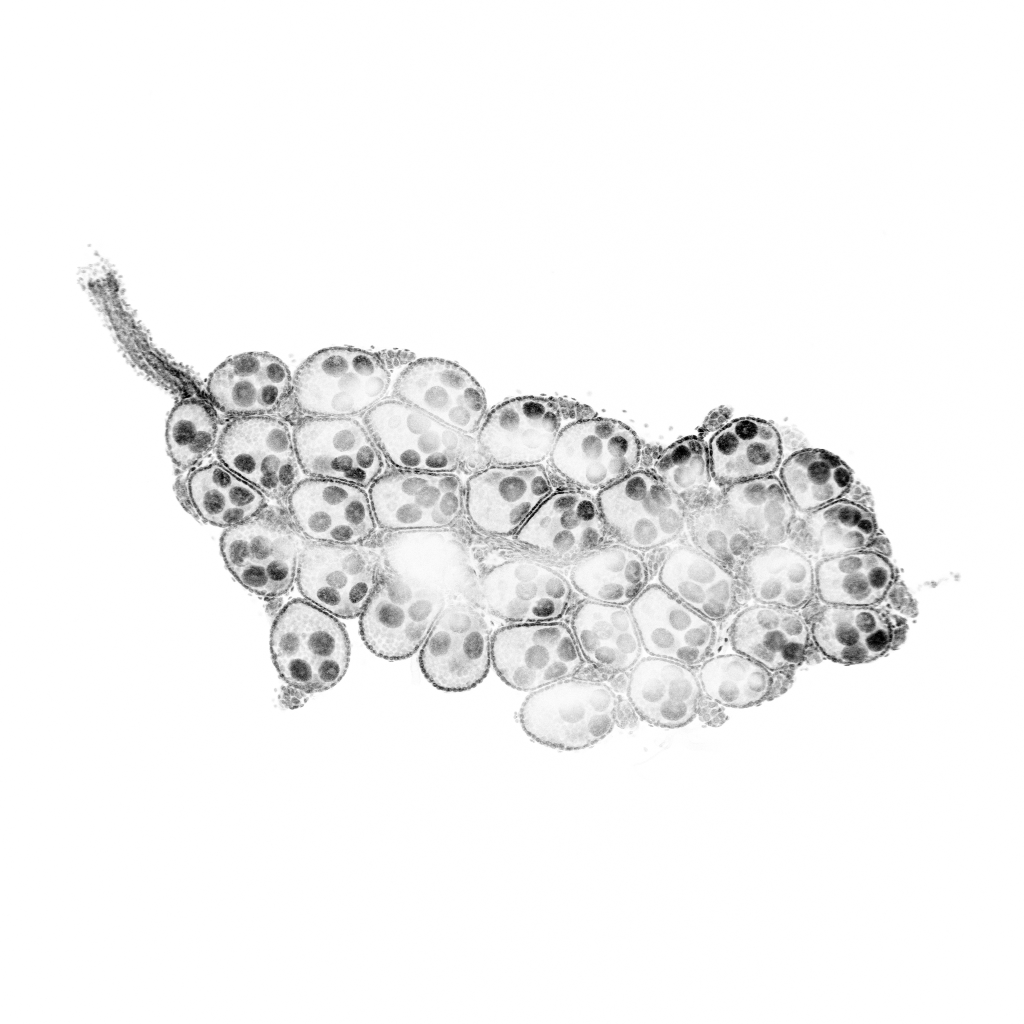

Supplement: Supplementary file 4 — Source data Fig. 2 [file 44319_2026_741_MOESM4_ESM.zip › Figure_2/2C/Aedes_aegypti_whole-ovary_DAPI.tif]

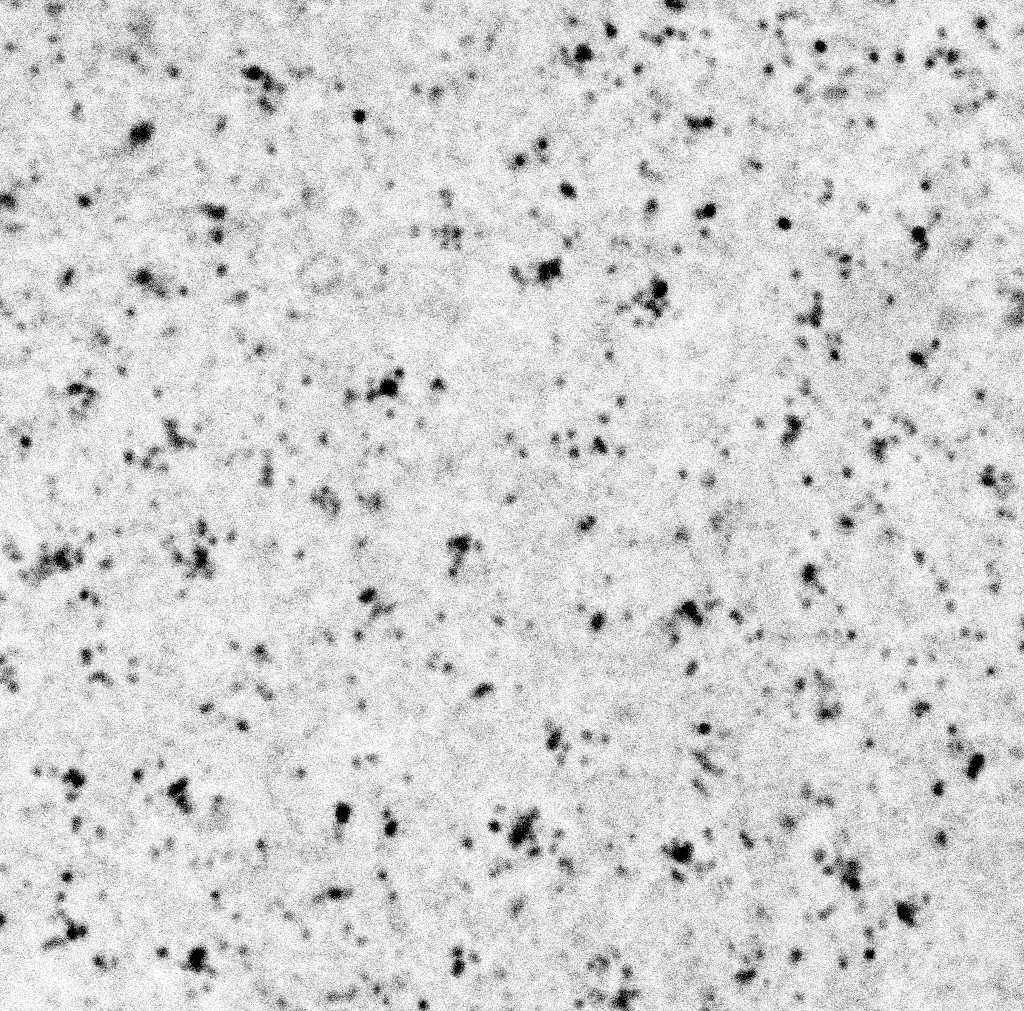

Supplement: Supplementary file 4 — Source data Fig. 2 [file 44319_2026_741_MOESM4_ESM.zip › Figure_2/2C/Aedes_aegypti_cluster3_RNA-FISH_birds-eye_FISH-only.tif]

Figure 2C

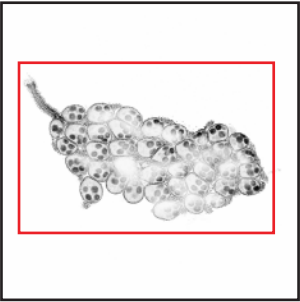

Supplement: Supplementary file 4 — Source data Fig. 2 [file 44319_2026_741_MOESM4_ESM.zip › Figure_2/2C/microscopy-images_cropping-information_Fig2C.pdf]

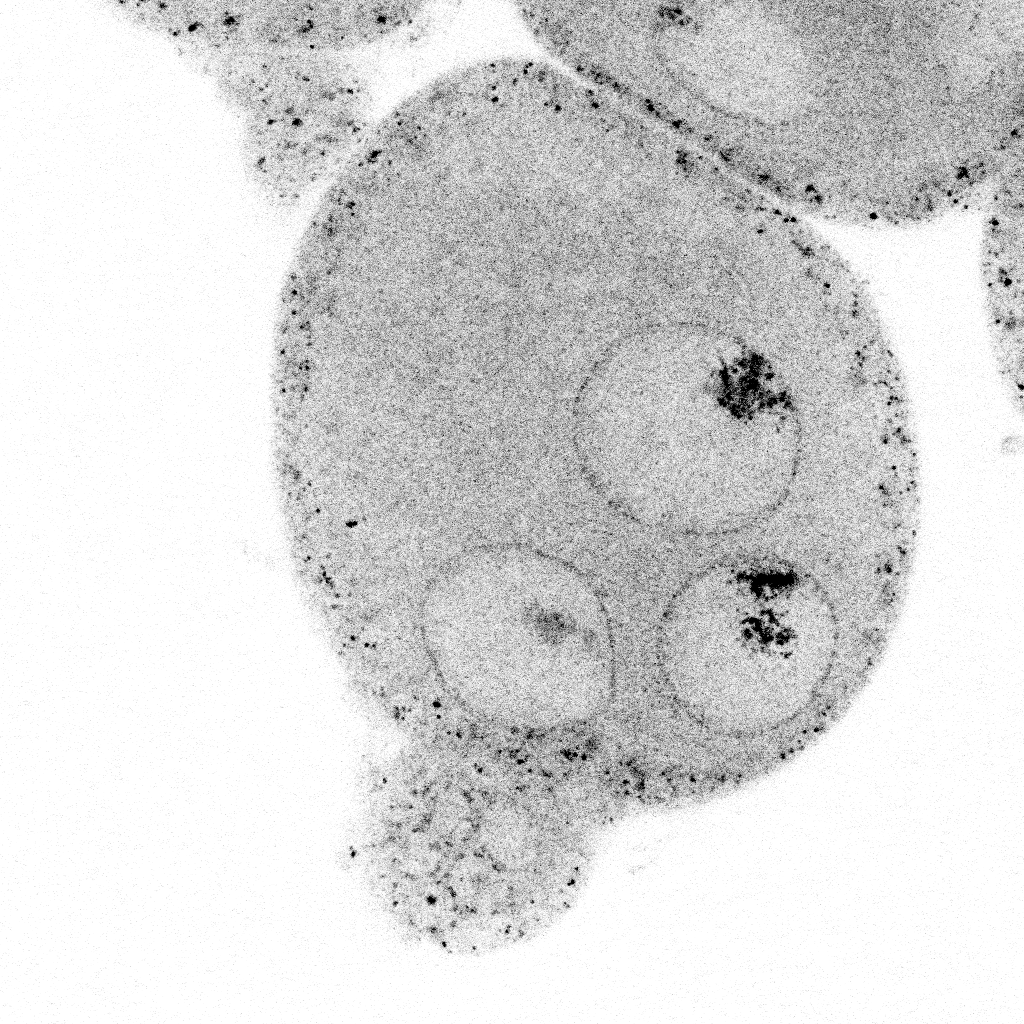

Supplement: Supplementary file 4 — Source data Fig. 2 [file 44319_2026_741_MOESM4_ESM.zip › Figure_2/2C/Aedes_aegypti_cluster3_RNA-FISH_FISH-only.tif]

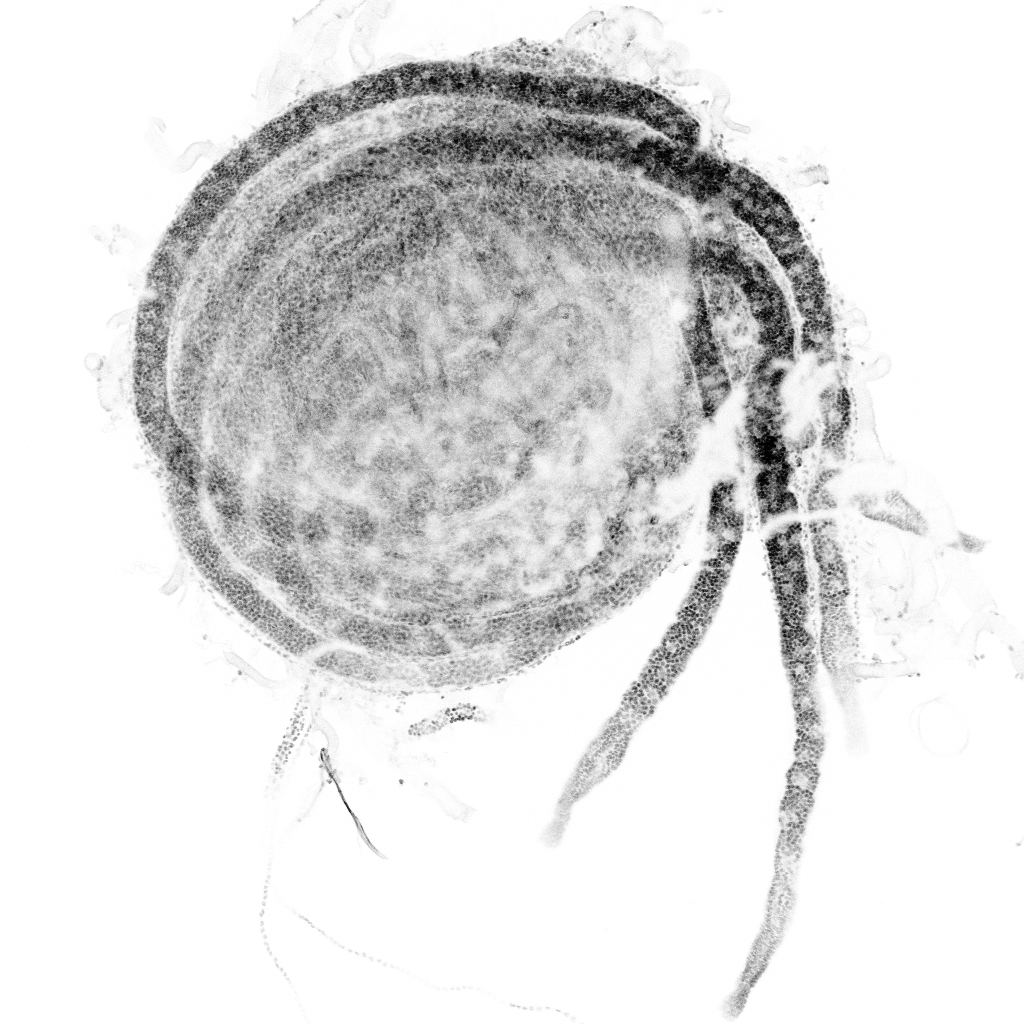

Supplement: Supplementary file 5 — Source data Fig. 3 [file 44319_2026_741_MOESM5_ESM.zip › Figure_3/3C/Tetragonula_carbonaria_whole-ovary_DAPI.tif]

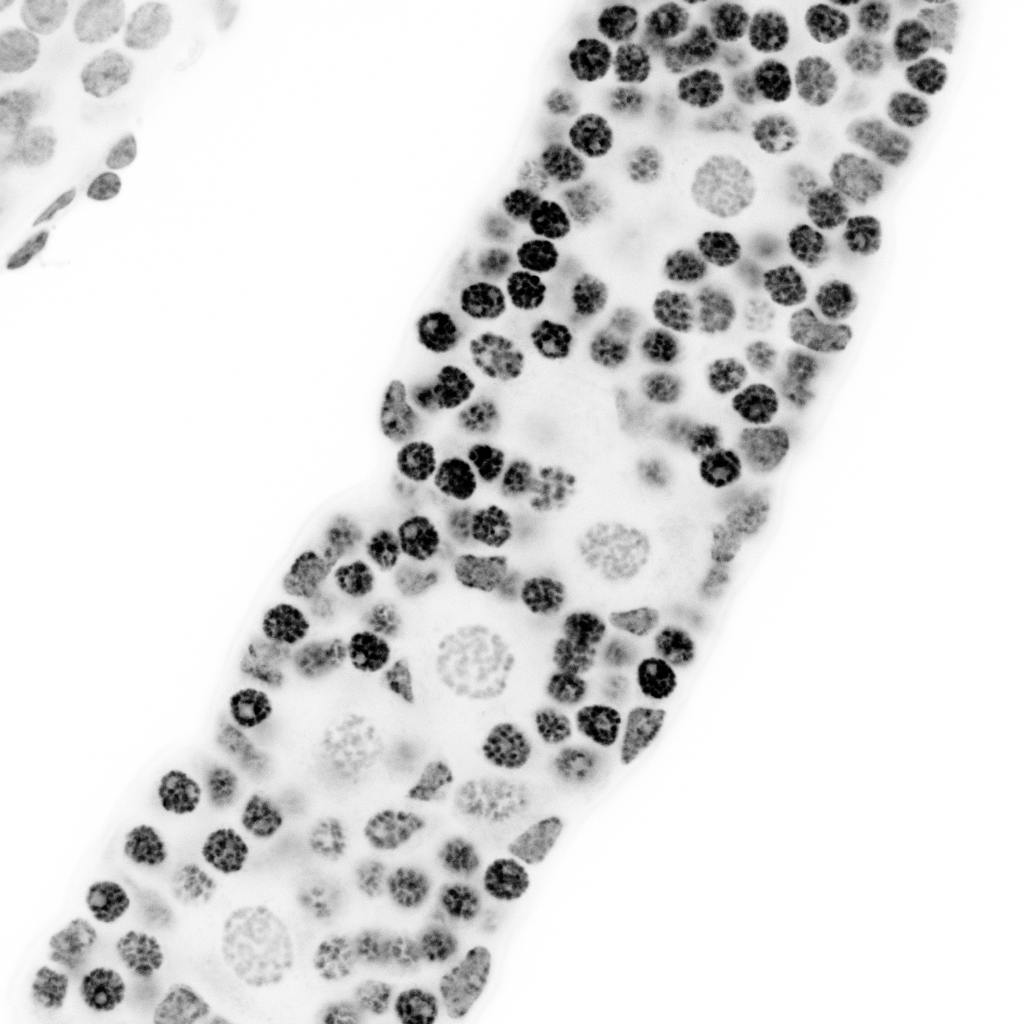

Supplement: Supplementary file 5 — Source data Fig. 3 [file 44319_2026_741_MOESM5_ESM.zip › Figure_3/3D/Tetragonula_carbonaria_cluster_RNA-FISH_DAPI-only.tif]

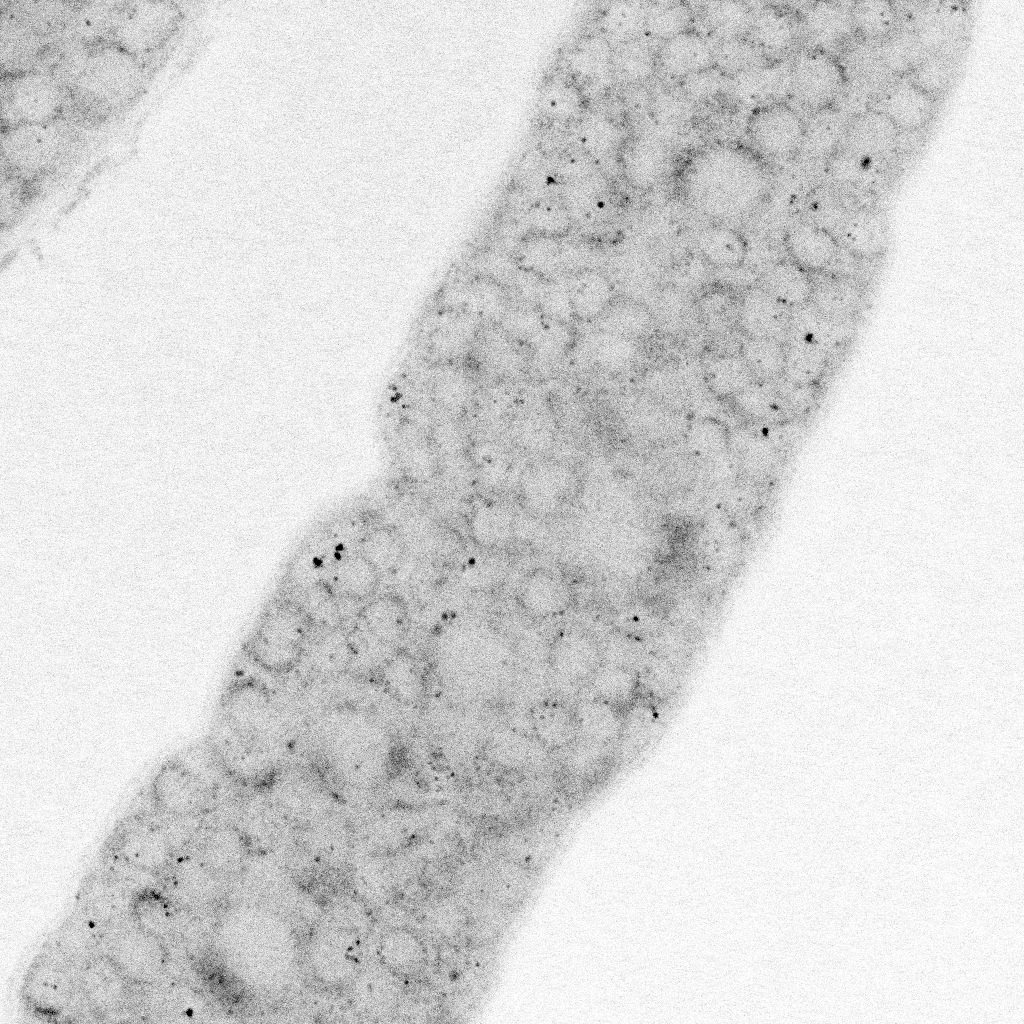

Supplement: Supplementary file 5 — Source data Fig. 3 [file 44319_2026_741_MOESM5_ESM.zip › Figure_3/3D/Tetragonula_carbonaria_cluster_RNA-FISH_FISH-only.tif]

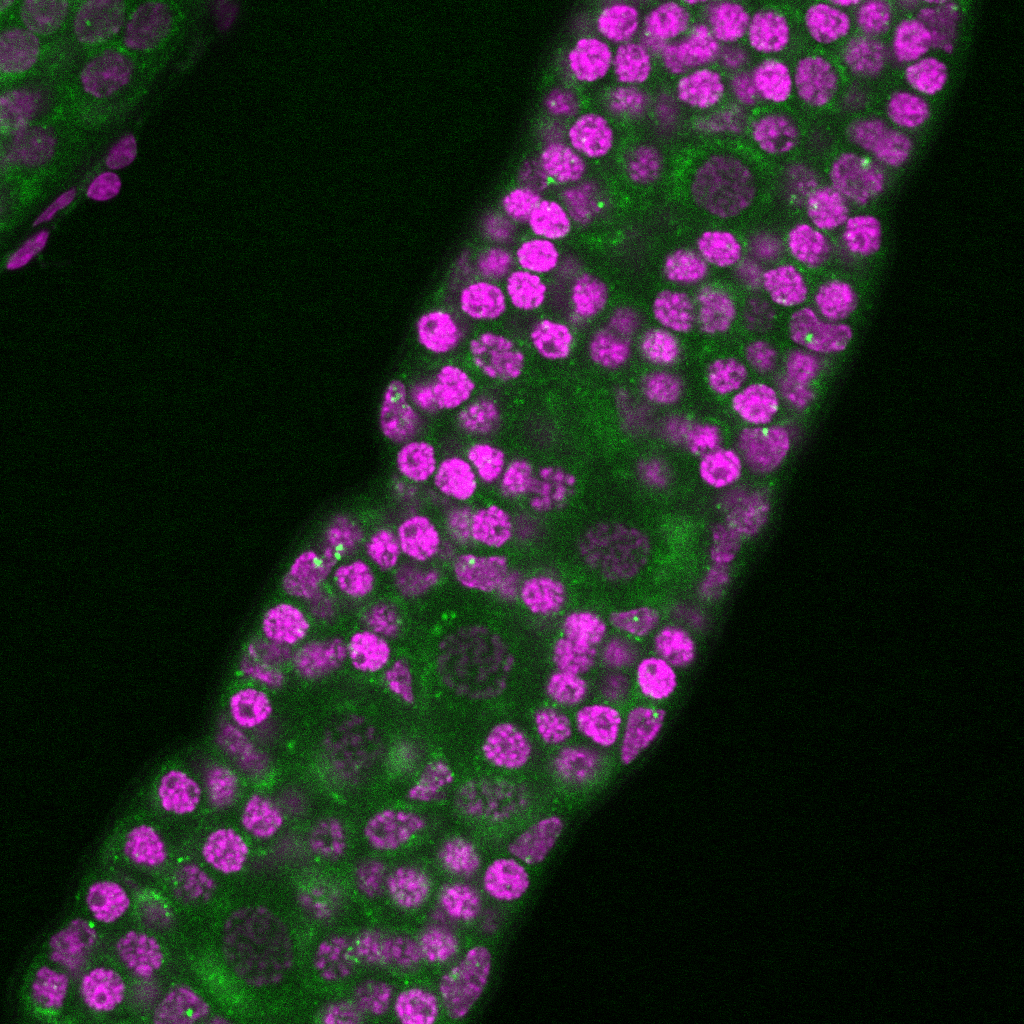

Supplement: Supplementary file 5 — Source data Fig. 3 [file 44319_2026_741_MOESM5_ESM.zip › Figure_3/3D/Tetragonula_carbonaria_cluster_RNA-FISH_FISH-DAPI.tif]

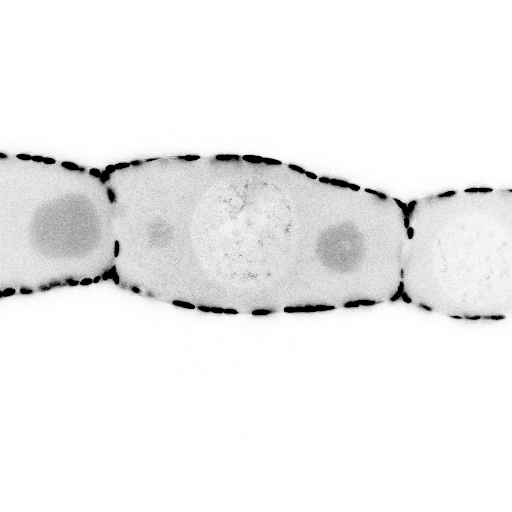

Supplement: Supplementary file 6 — Source data Fig. 4 [file 44319_2026_741_MOESM6_ESM.zip › Figure_4/4E/Acheta_domesticus_mid-stages_cluster_RNA-FISH_DAPI-only.tif]

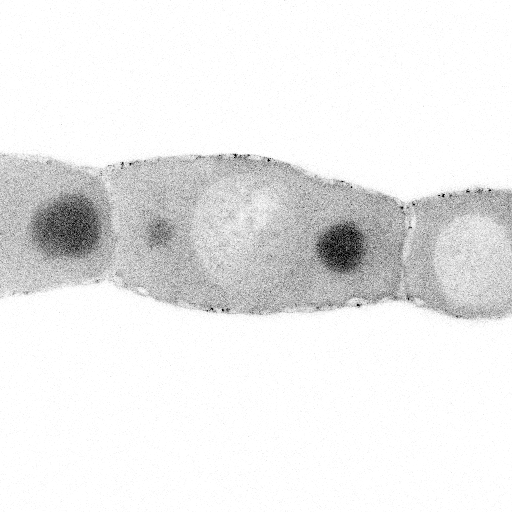

Supplement: Supplementary file 6 — Source data Fig. 4 [file 44319_2026_741_MOESM6_ESM.zip › Figure_4/4E/Acheta_domesticus_mid-stages_cluster_RNA-FISH_FISH-only.tif]

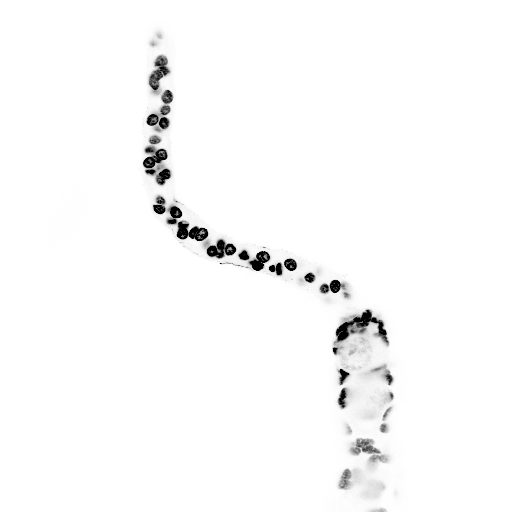

Supplement: Supplementary file 6 — Source data Fig. 4 [file 44319_2026_741_MOESM6_ESM.zip › Figure_4/4E/Acheta_domesticus_germarium_cluster_RNA-FISH_DAPI-only.tif]

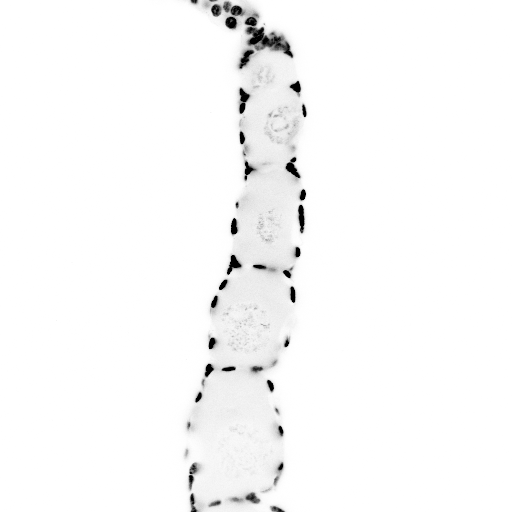

Supplement: Supplementary file 6 — Source data Fig. 4 [file 44319_2026_741_MOESM6_ESM.zip › Figure_4/4E/Acheta_domesticus_early-stages_cluster_RNA-FISH_DAPI-only.tif]

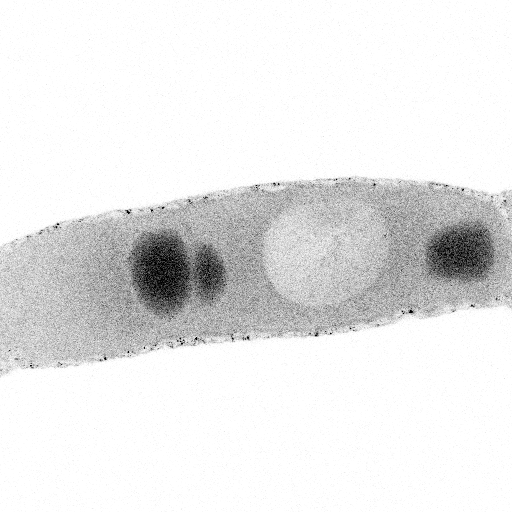

Supplement: Supplementary file 6 — Source data Fig. 4 [file 44319_2026_741_MOESM6_ESM.zip › Figure_4/4E/Acheta_domesticus_late-stage_cluster_RNA-FISH_FISH-only.tif]

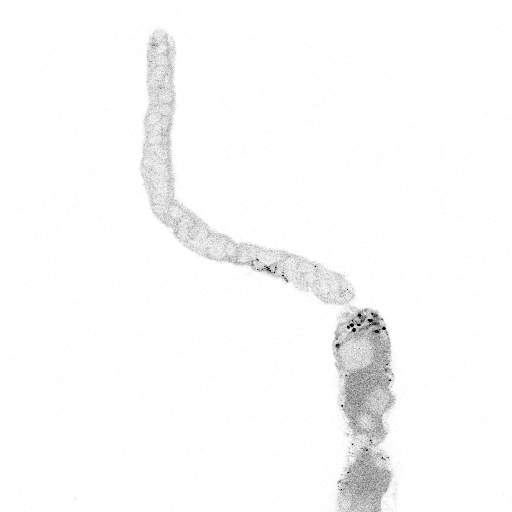

Supplement: Supplementary file 6 — Source data Fig. 4 [file 44319_2026_741_MOESM6_ESM.zip › Figure_4/4E/Acheta_domesticus_germarium_cluster_RNA-FISH_FISH-only.tif]

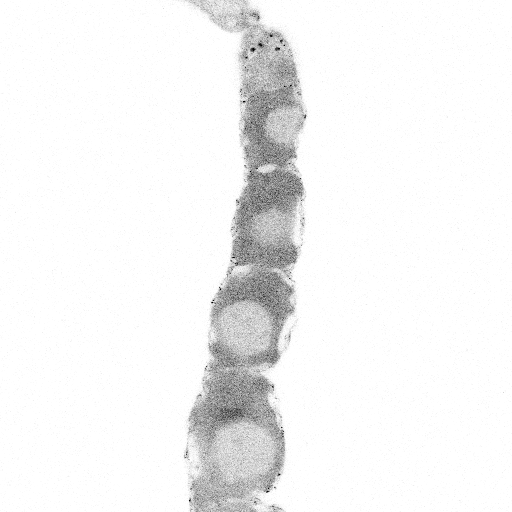

Supplement: Supplementary file 6 — Source data Fig. 4 [file 44319_2026_741_MOESM6_ESM.zip › Figure_4/4E/Acheta_domesticus_early-stages_cluster_RNA-FISH_FISH-only.tif]

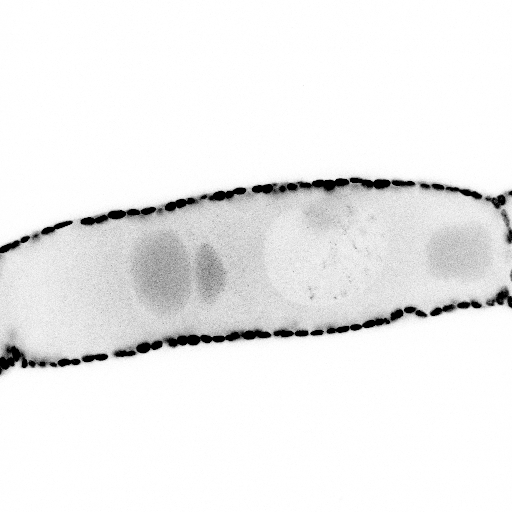

Supplement: Supplementary file 6 — Source data Fig. 4 [file 44319_2026_741_MOESM6_ESM.zip › Figure_4/4E/Acheta_domesticus_late-stage_cluster_RNA-FISH_DAPI-only.tif]

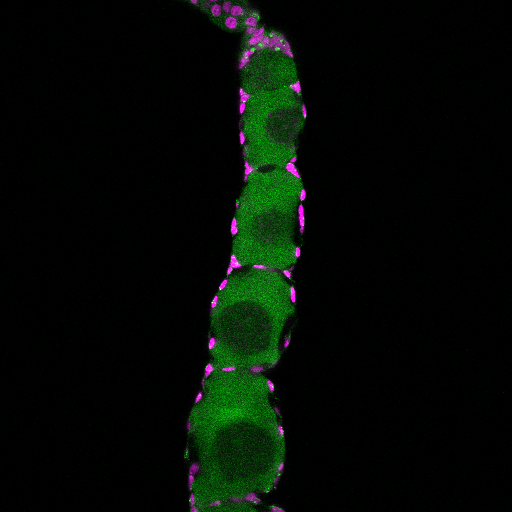

Supplement: Supplementary file 6 — Source data Fig. 4 [file 44319_2026_741_MOESM6_ESM.zip › Figure_4/4E/Acheta_domesticus_early-stages_cluster_RNA-FISH_FISH-DAPI.tif]

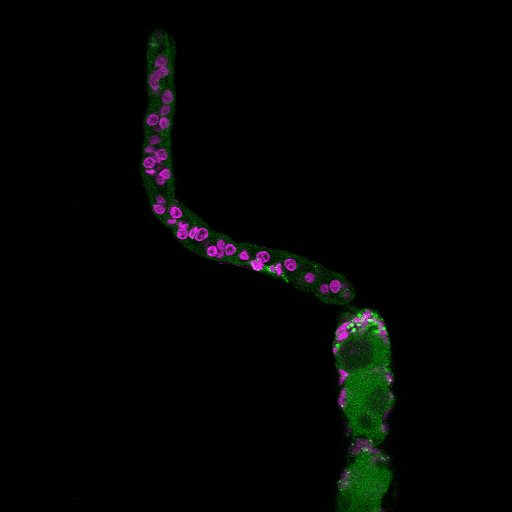

Supplement: Supplementary file 6 — Source data Fig. 4 [file 44319_2026_741_MOESM6_ESM.zip › Figure_4/4E/Acheta_domesticus_germarium_cluster_RNA-FISH_FISH-DAPI.tif]

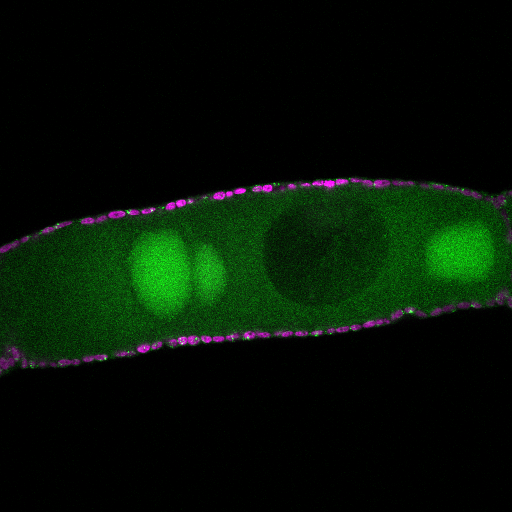

Supplement: Supplementary file 6 — Source data Fig. 4 [file 44319_2026_741_MOESM6_ESM.zip › Figure_4/4E/Acheta_domesticus_late-stage_cluster_RNA-FISH_FISH-DAPI.tif]

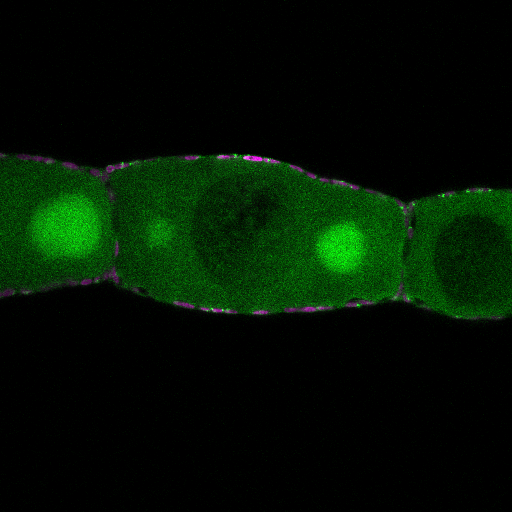

Supplement: Supplementary file 6 — Source data Fig. 4 [file 44319_2026_741_MOESM6_ESM.zip › Figure_4/4E/Acheta_domesticus_mid-stages_cluster_RNA-FISH_FISH-DAPI.tif]

Figure 4E

germarium

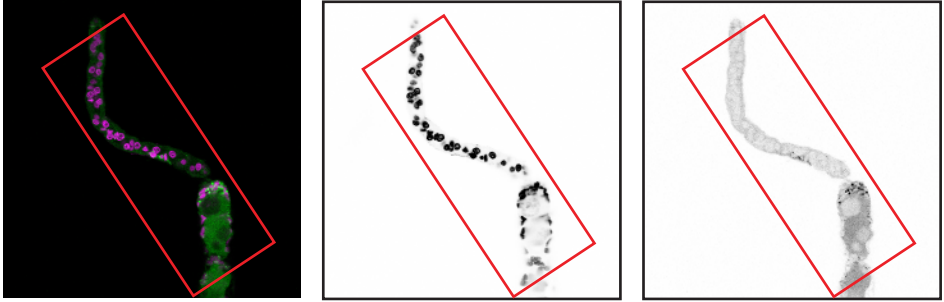

early stages

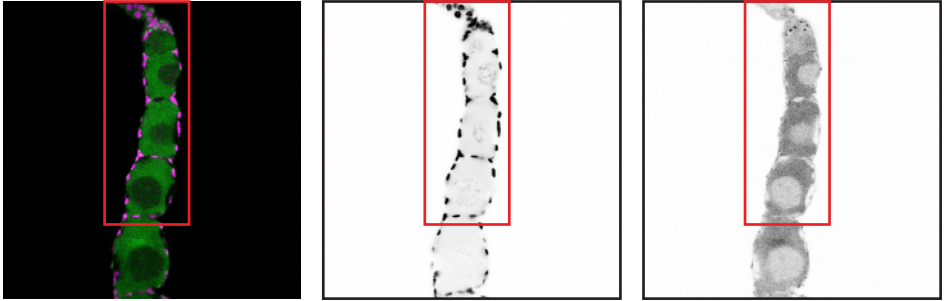

mid stages

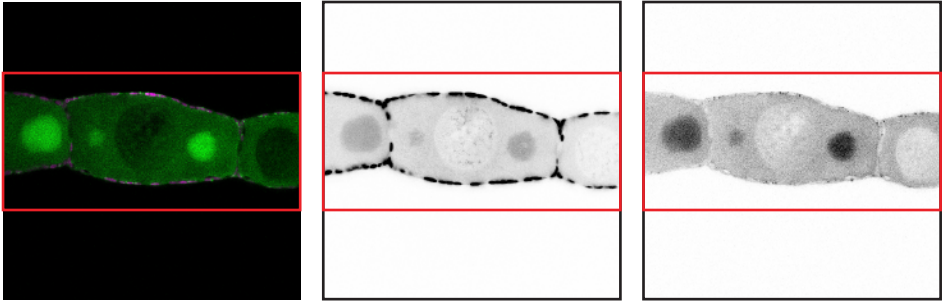

late stage

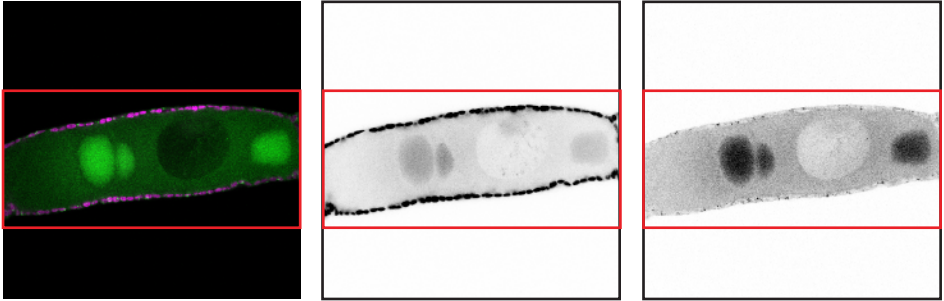

Supplement: Supplementary file 6 — Source data Fig. 4 [file 44319_2026_741_MOESM6_ESM.zip › Figure_4/4E/microscopy-images_cropping-information_Fig4E.pdf]

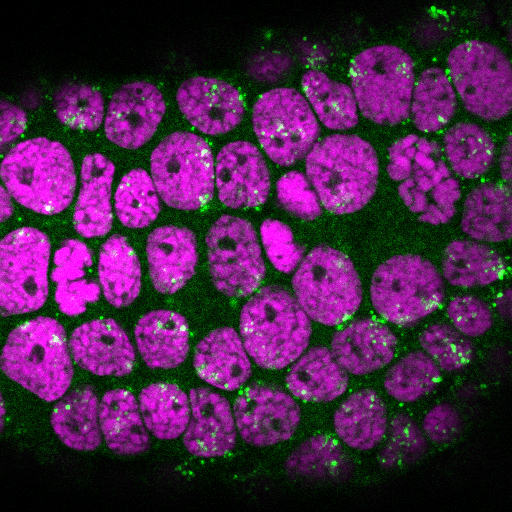

Supplement: Supplementary file 6 — Source data Fig. 4 [file 44319_2026_741_MOESM6_ESM.zip › Figure_4/4F/Acheta_domesticus_mid-stage_RNA-FISH_birds-eye_FISH-DAPI.tif]

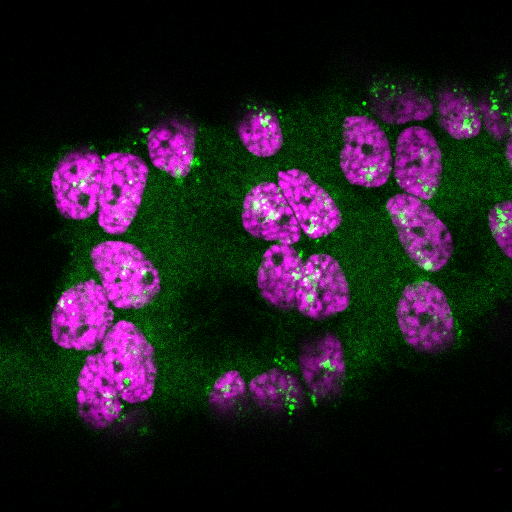

Supplement: Supplementary file 6 — Source data Fig. 4 [file 44319_2026_741_MOESM6_ESM.zip › Figure_4/4F/Acheta_domesticus_early-stage_RNA-FISH_birds-eye_FISH-DAPI.tif]

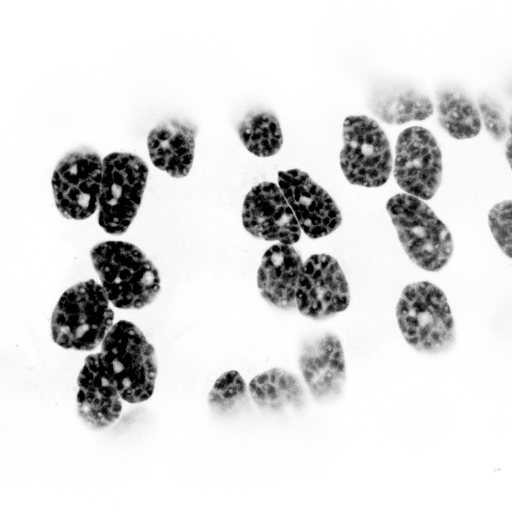

Supplement: Supplementary file 6 — Source data Fig. 4 [file 44319_2026_741_MOESM6_ESM.zip › Figure_4/4F/Acheta_domesticus_early-stage_RNA-FISH_birds-eye_DAPI-only.tif]

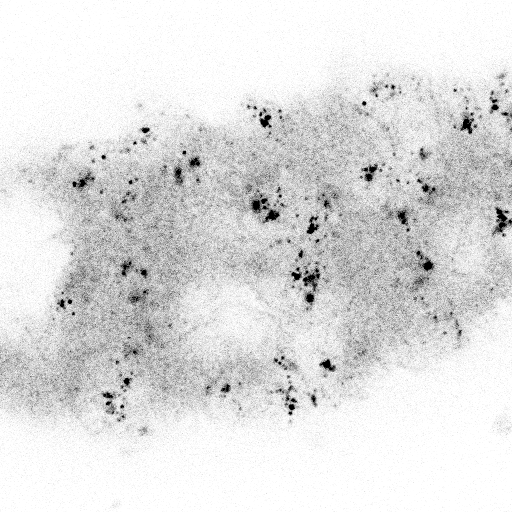

Supplement: Supplementary file 6 — Source data Fig. 4 [file 44319_2026_741_MOESM6_ESM.zip › Figure_4/4F/Acheta_domesticus_early-stage_RNA-FISH_birds-eye_FISH-only.tif]

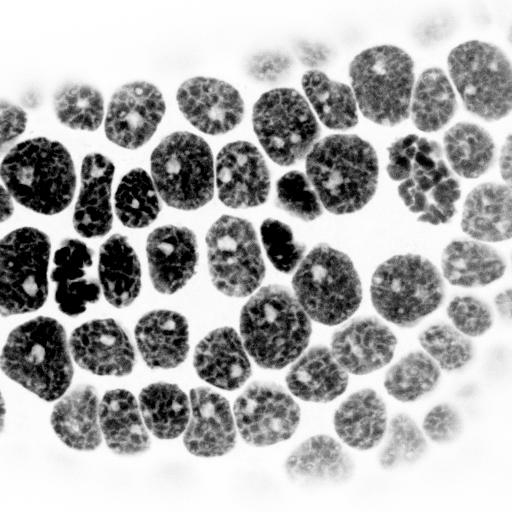

Supplement: Supplementary file 6 — Source data Fig. 4 [file 44319_2026_741_MOESM6_ESM.zip › Figure_4/4F/Acheta_domesticus_mid-stage_RNA-FISH_birds-eye_DAPI-only.tif]

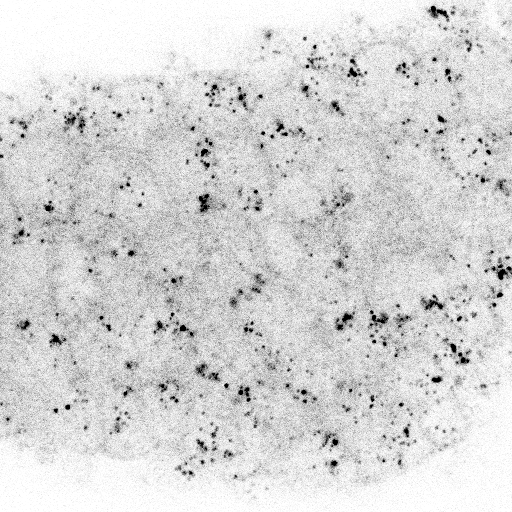

Supplement: Supplementary file 6 — Source data Fig. 4 [file 44319_2026_741_MOESM6_ESM.zip › Figure_4/4F/Acheta_domesticus_mid-stage_RNA-FISH_birds-eye_FISH-only.tif]

150 —  
100 —  
75 —  
50 —  
37 —  
25 —  
kDa

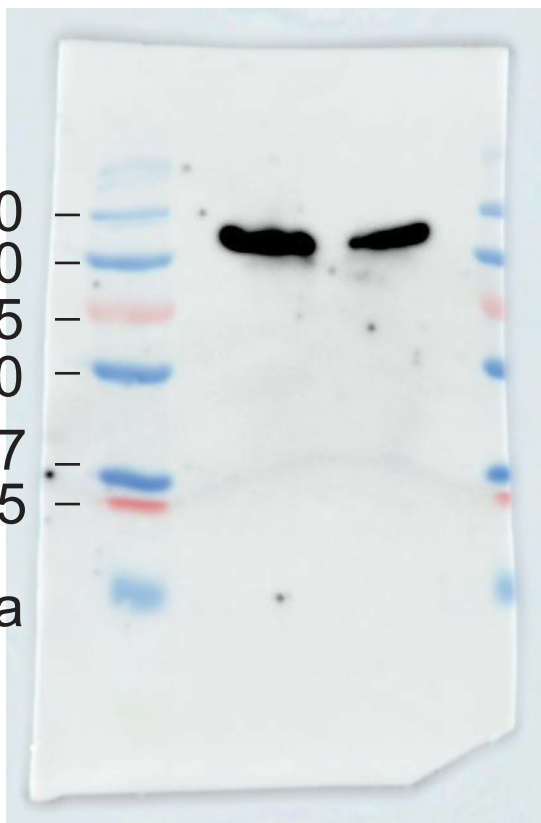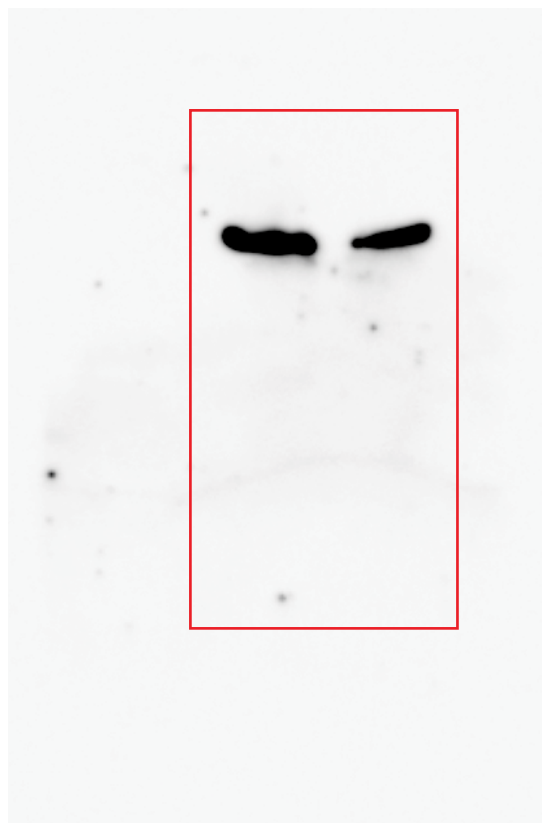

Supplement: Supplementary file 7 — Source data Fig. 5 [file 44319_2026_741_MOESM7_ESM.zip › Figure_5/5B'/Anopheles_stephensi_Aub-like_western-blotting_cropping-information.pdf]

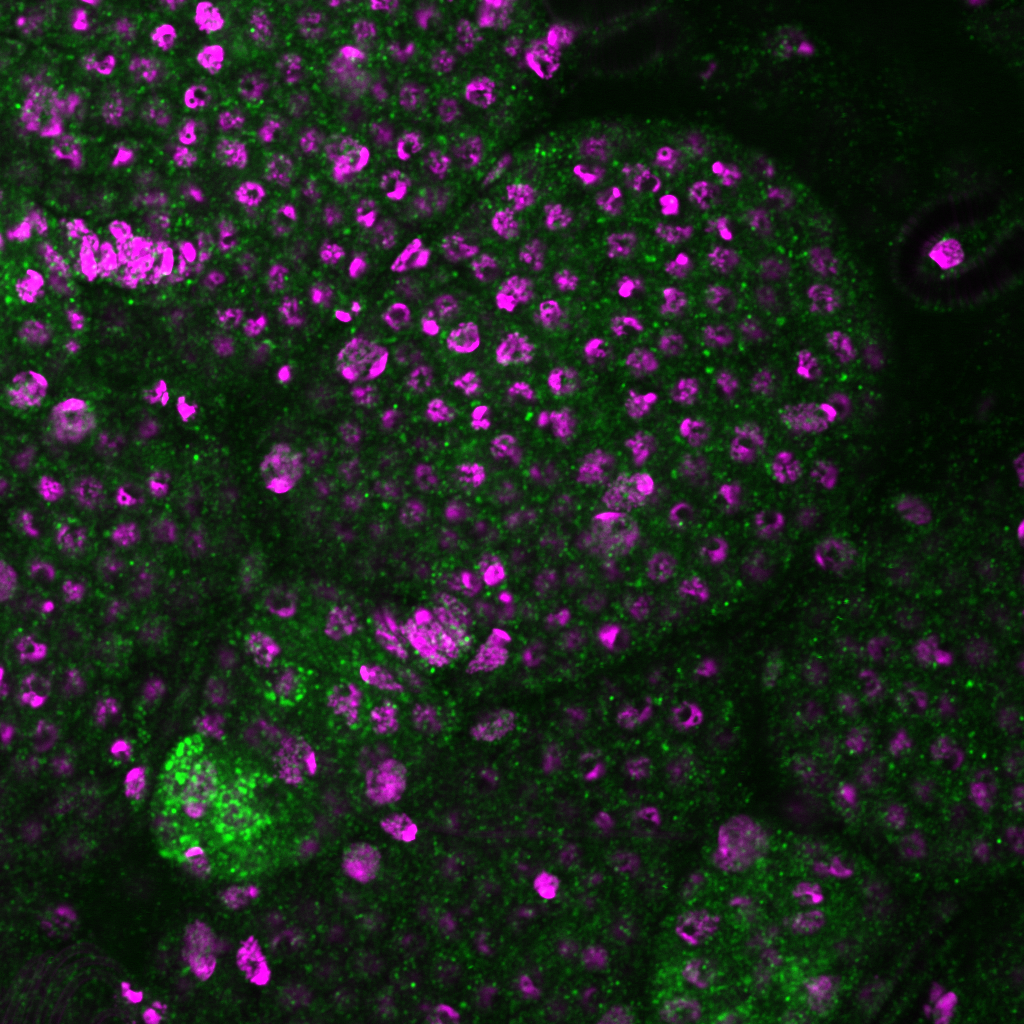

Supplement: Supplementary file 7 — Source data Fig. 5 [file 44319_2026_741_MOESM7_ESM.zip › Figure_5/5B/Anopheles_stephensi_Aub-like_IF_birds-eye_IF-DAPI.tif]

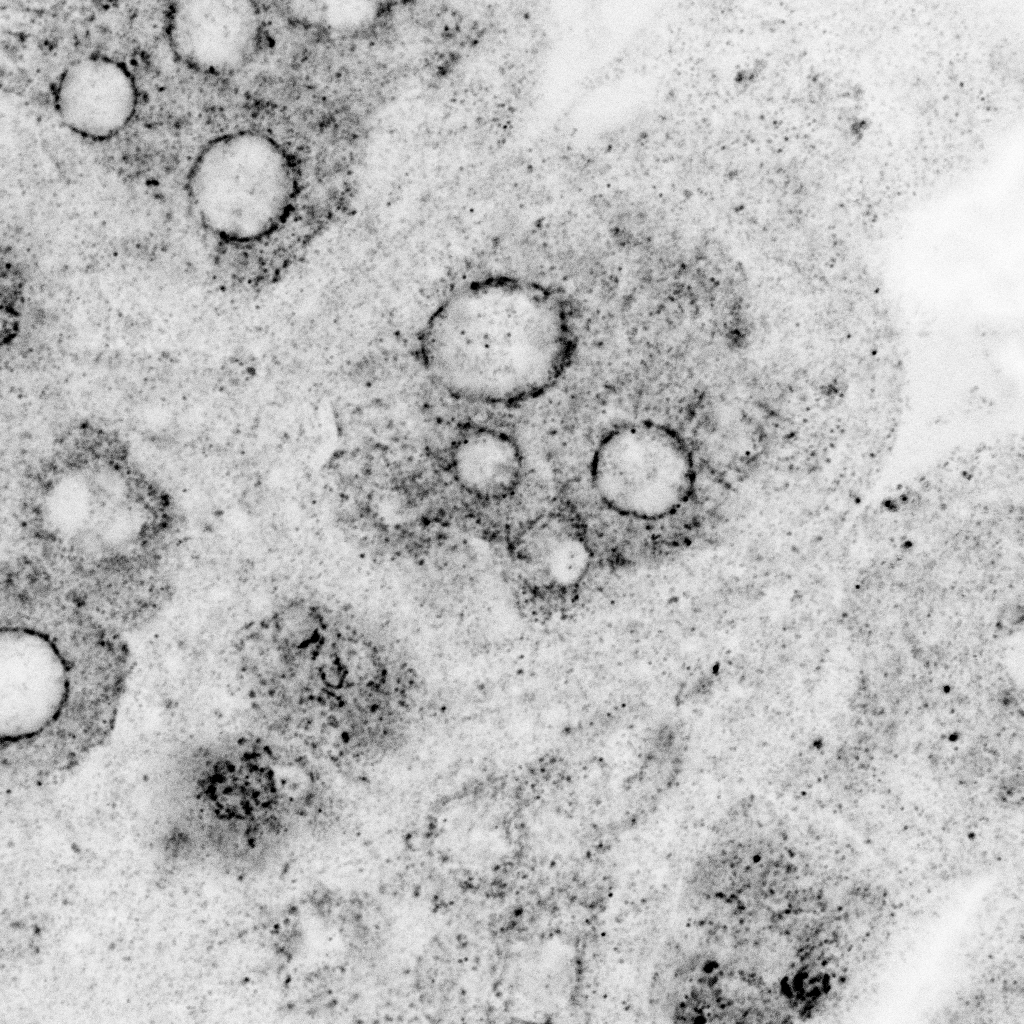

Supplement: Supplementary file 7 — Source data Fig. 5 [file 44319_2026_741_MOESM7_ESM.zip › Figure_5/5B/Anopheles_stephensi_Aub-like_IF_IF-only.tif]

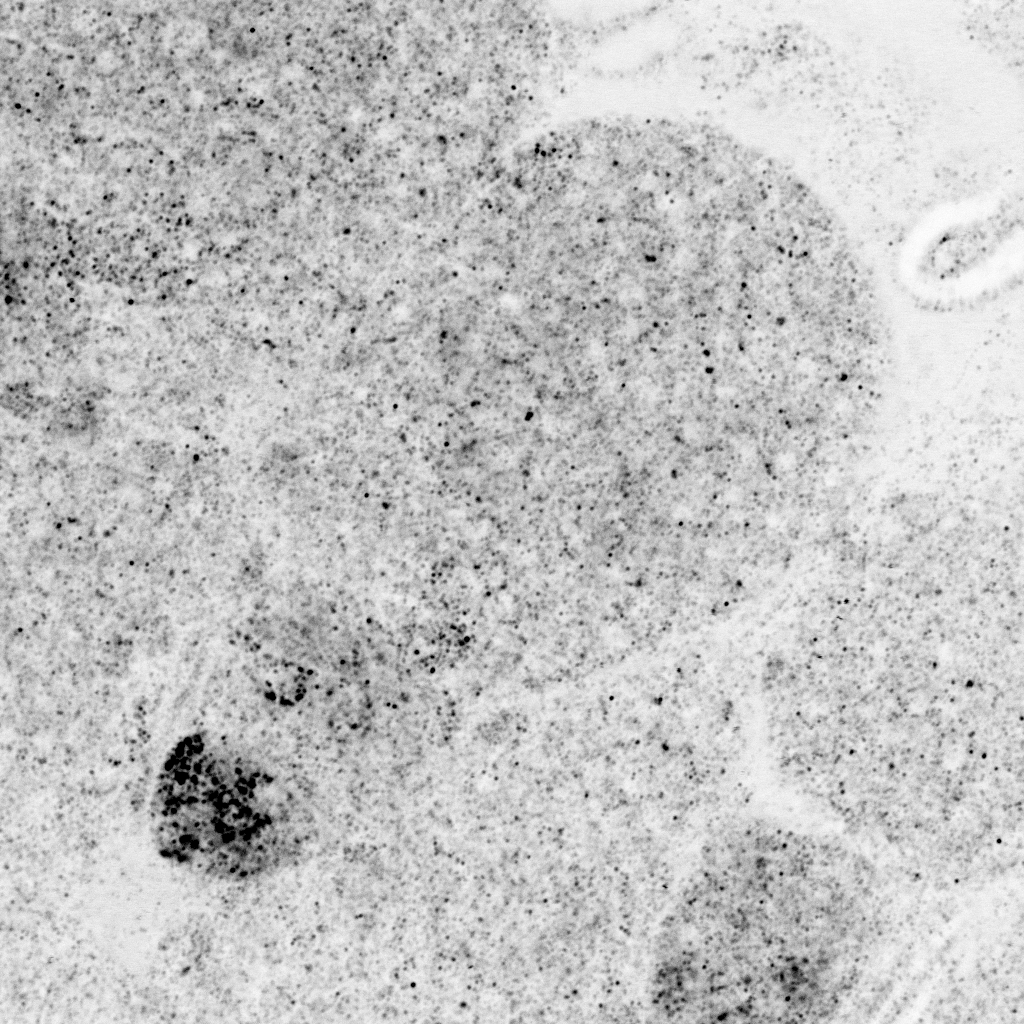

Supplement: Supplementary file 7 — Source data Fig. 5 [file 44319_2026_741_MOESM7_ESM.zip › Figure_5/5B/Anopheles_stephensi_Aub-like_IF_birds-eye_IF-only.tif]

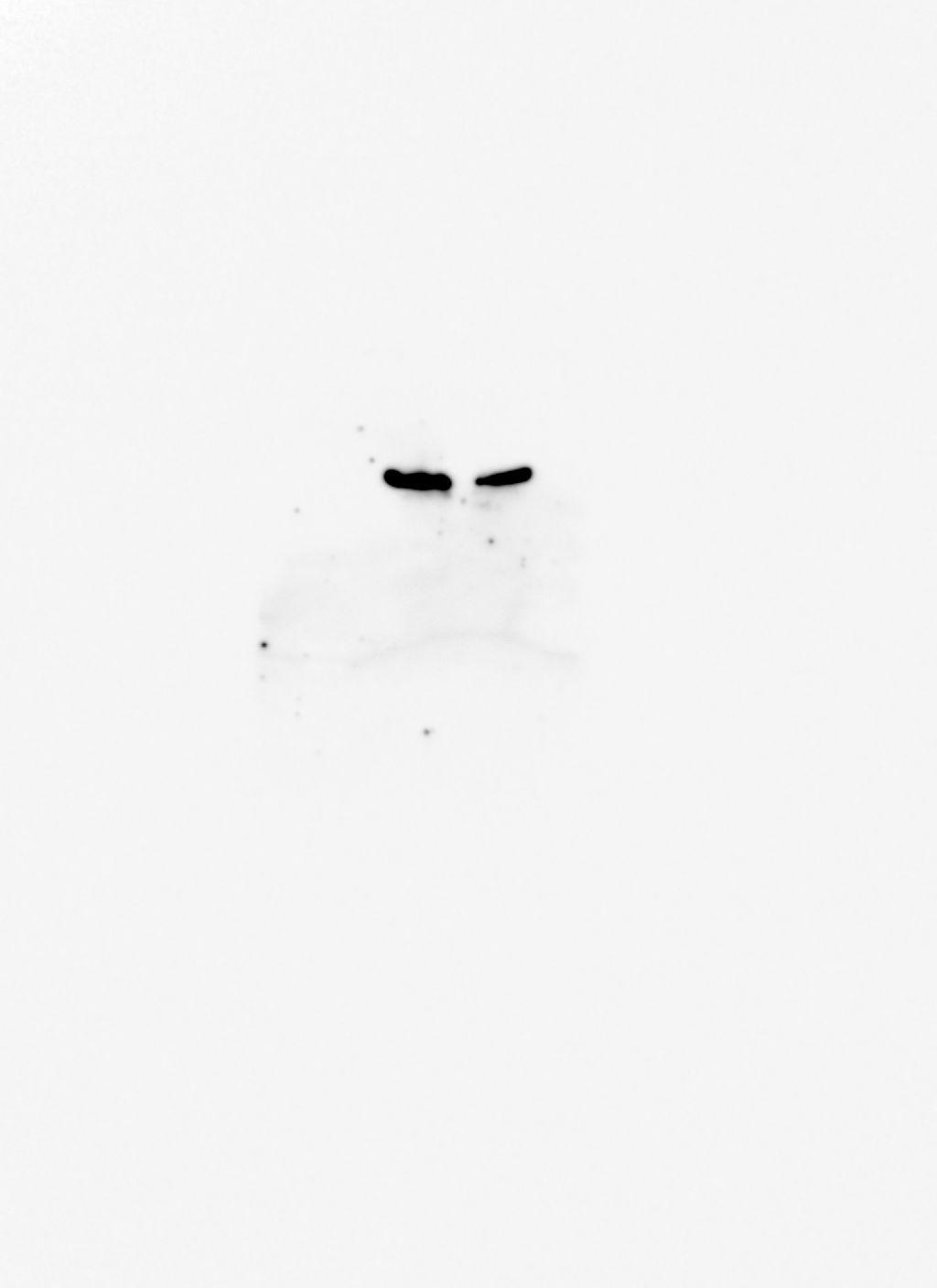

Supplement: Supplementary file 7 — Source data Fig. 5 [file 44319_2026_741_MOESM7_ESM.zip › Figure_5/5B/Anopheles_stephensi_Aub-like_western-blotting_raw.tif]

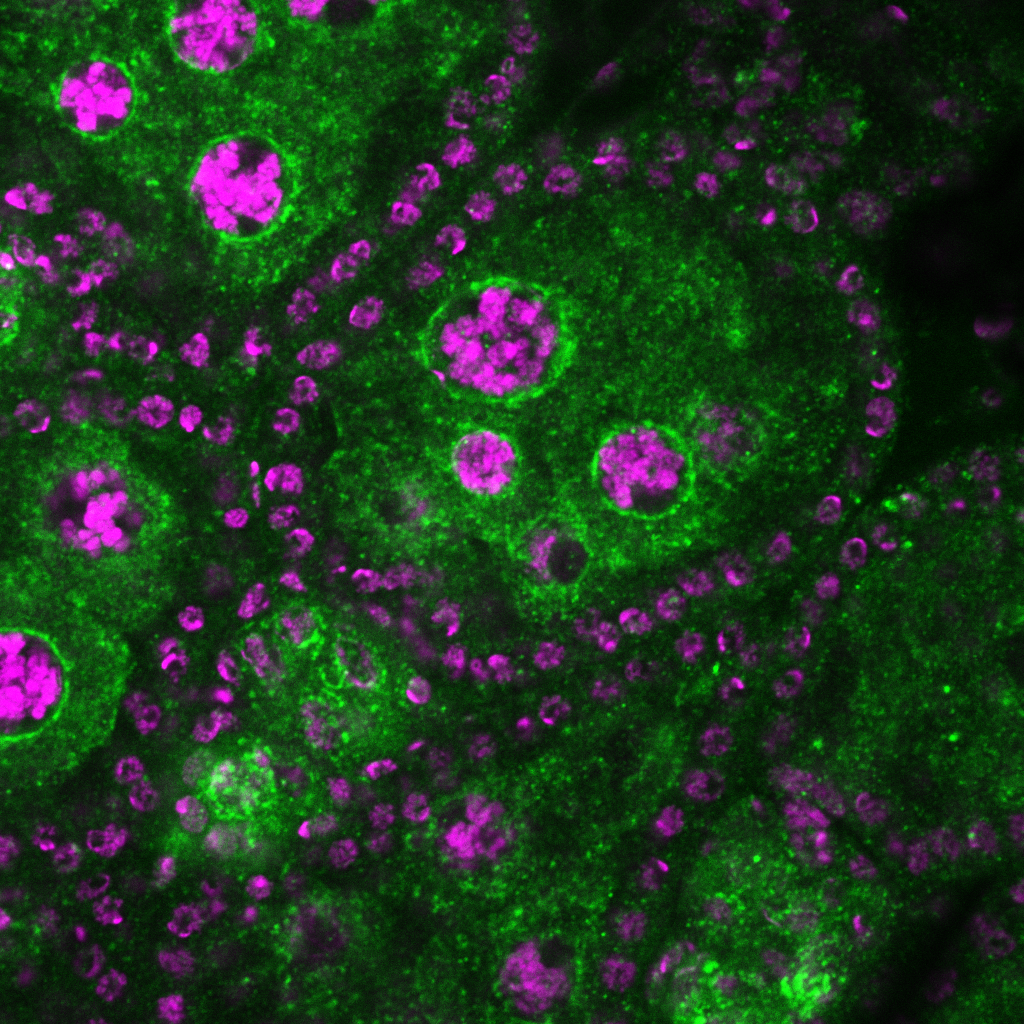

Supplement: Supplementary file 7 — Source data Fig. 5 [file 44319_2026_741_MOESM7_ESM.zip › Figure_5/5B/Anopheles_stephensi_Aub-like_IF_IF-DAPI.tif]

Figure 5B

Images were flipped upside down from the original images.

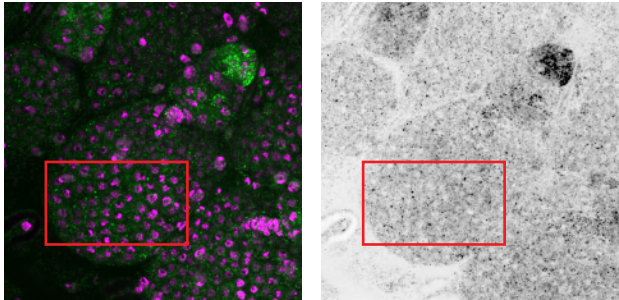

Supplement: Supplementary file 7 — Source data Fig. 5 [file 44319_2026_741_MOESM7_ESM.zip › Figure_5/5B/microscopy-images_cropping-information_Fig5B.pdf]

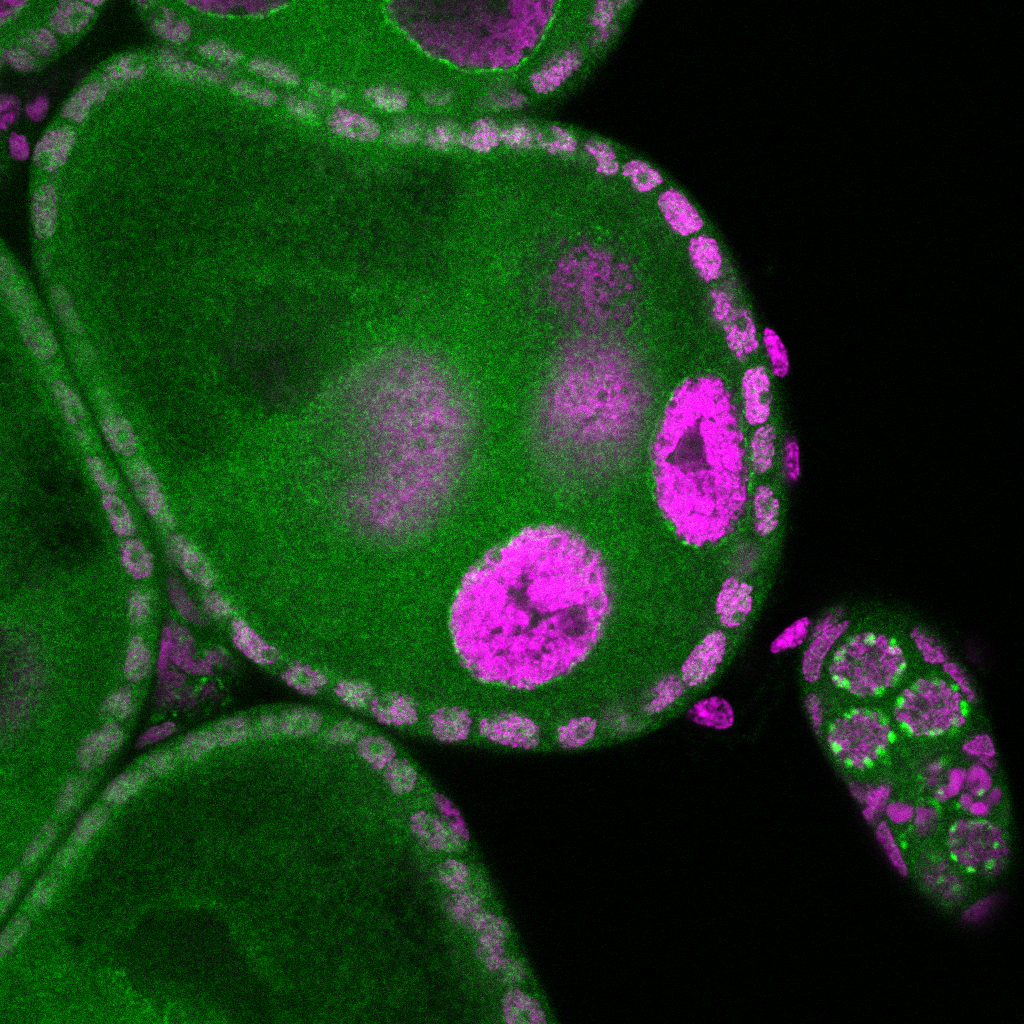

Supplement: Supplementary file 8 — Figure EV2 Source Data [file 44319_2026_741_MOESM8_ESM.zip › Figure_EV2/EV2C/Aedes_aegypti_control_RNA-FISH_FISH-DAPI.tif]

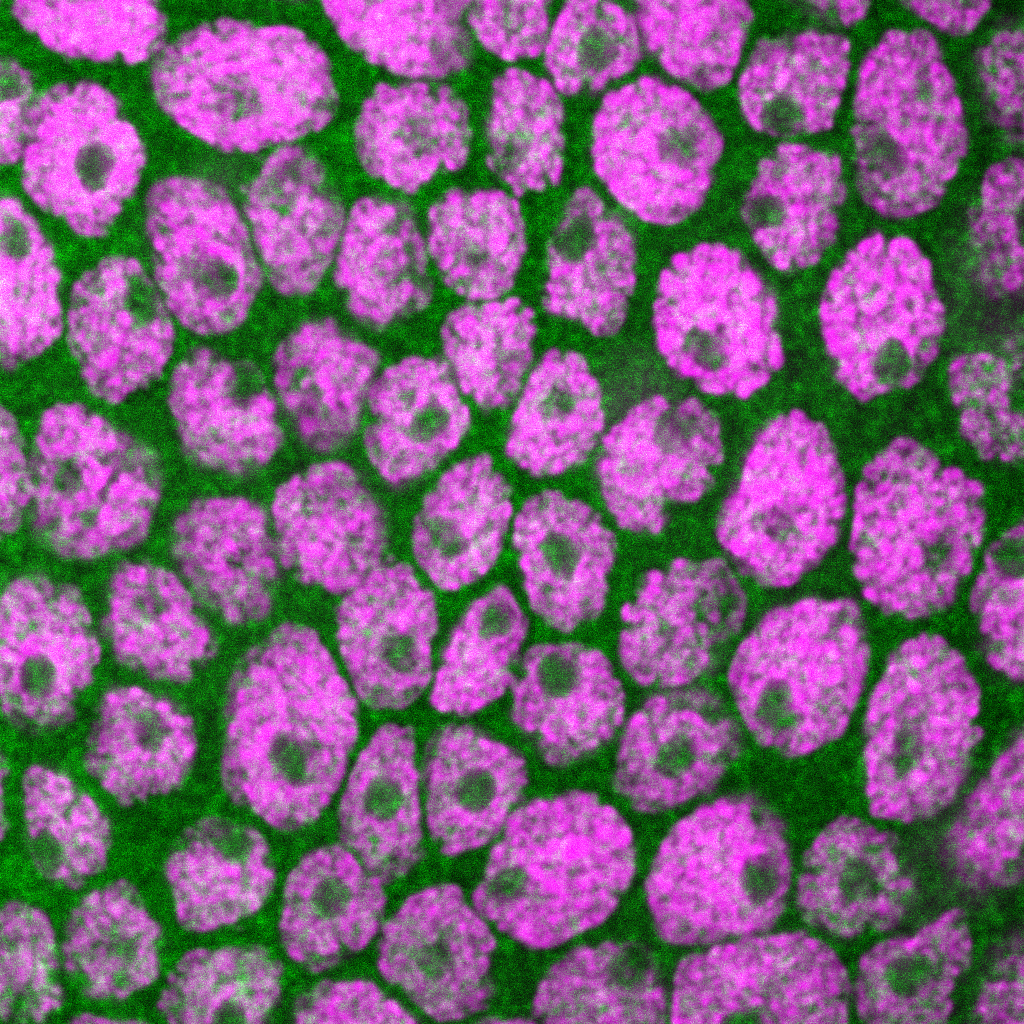

Supplement: Supplementary file 8 — Figure EV2 Source Data [file 44319_2026_741_MOESM8_ESM.zip › Figure_EV2/EV2C/Aedes_aegypti_control_RNA-FISH_birds-eye_FISH-DAPI.tif]

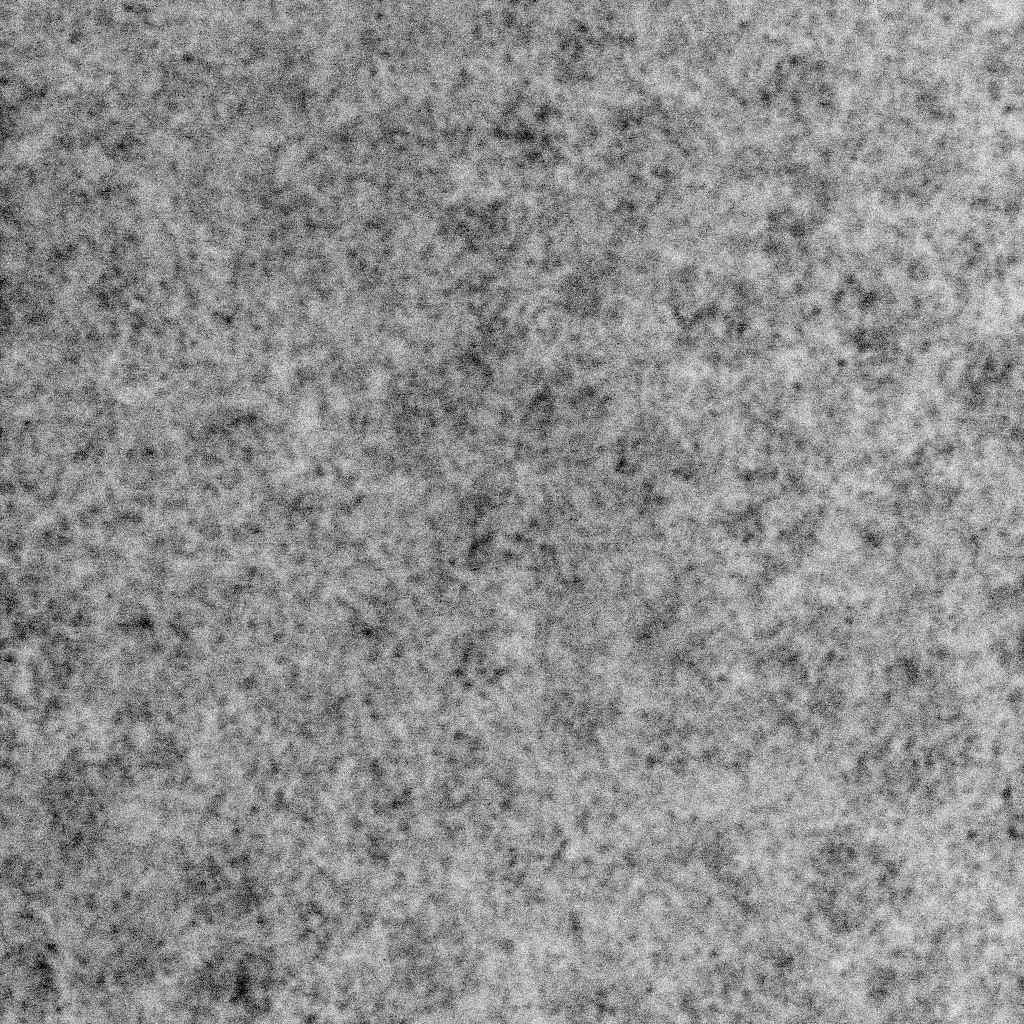

Supplement: Supplementary file 8 — Figure EV2 Source Data [file 44319_2026_741_MOESM8_ESM.zip › Figure_EV2/EV2C/Aedes_aegypti_control_RNA-FISH_birds-eye_FISH-only.tif]

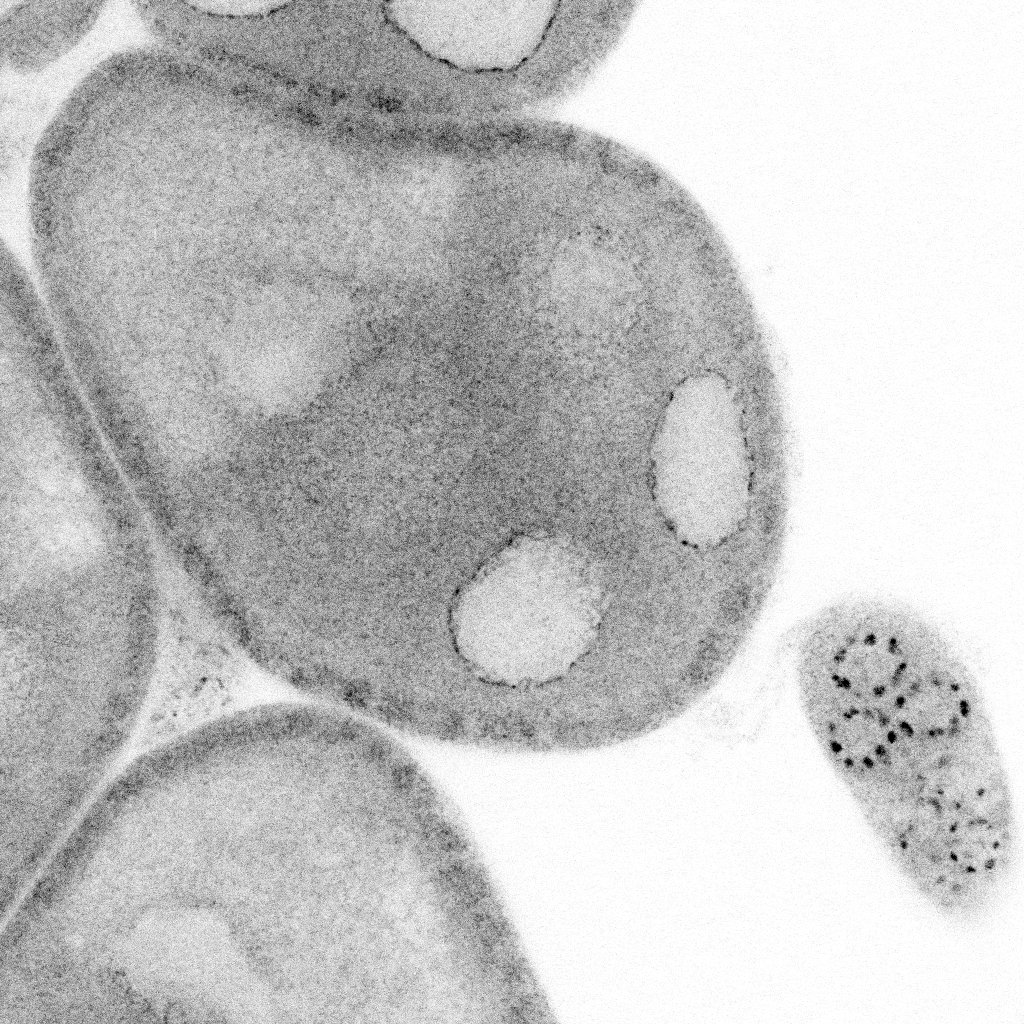

Supplement: Supplementary file 8 — Figure EV2 Source Data [file 44319_2026_741_MOESM8_ESM.zip › Figure_EV2/EV2C/Aedes_aegypti_control_RNA-FISH_FISH-only.tif]

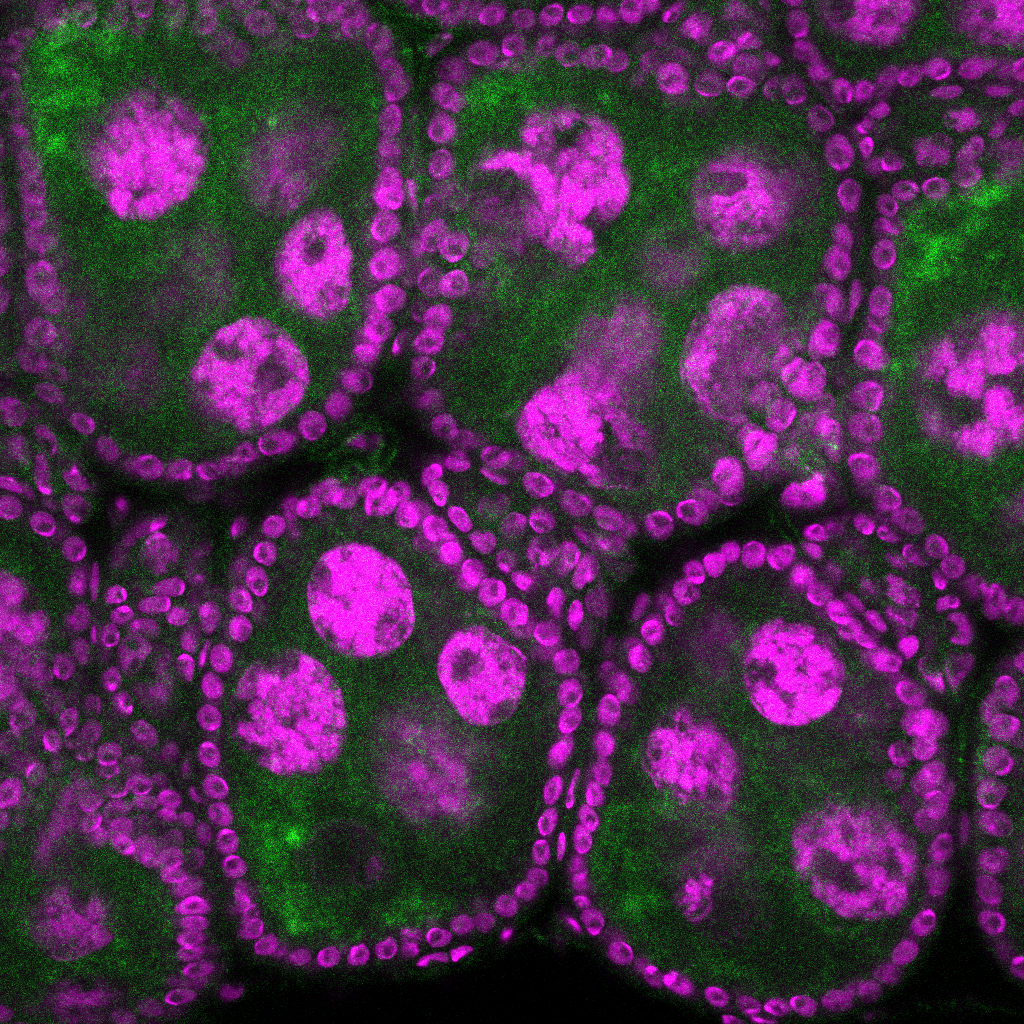

Supplement: Supplementary file 8 — Figure EV2 Source Data [file 44319_2026_741_MOESM8_ESM.zip › Figure_EV2/EV2I/Anopheles_stephensi_control_RNA-FISH_FISH-DAPI.tif]

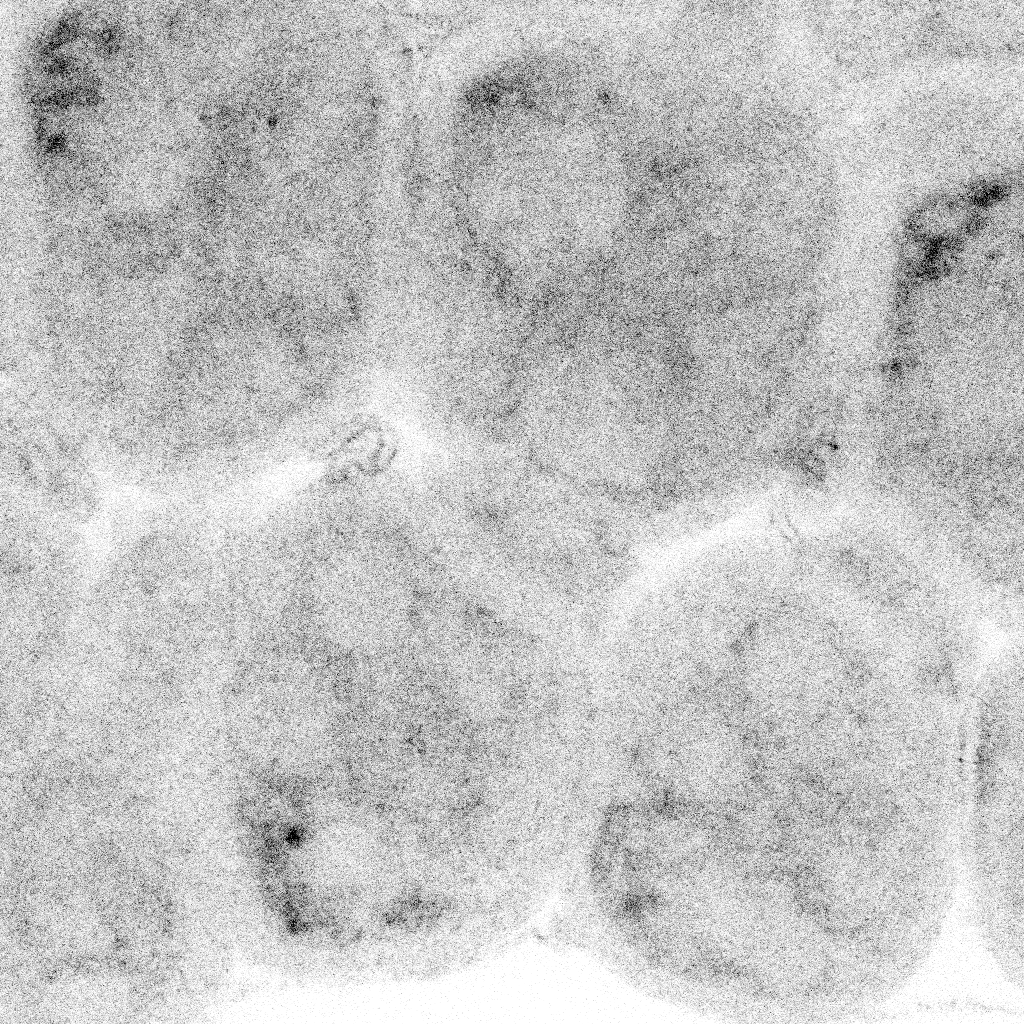

Supplement: Supplementary file 8 — Figure EV2 Source Data [file 44319_2026_741_MOESM8_ESM.zip › Figure_EV2/EV2I/Anopheles_stephensi_control_RNA-FISH_FISH-only.tif]

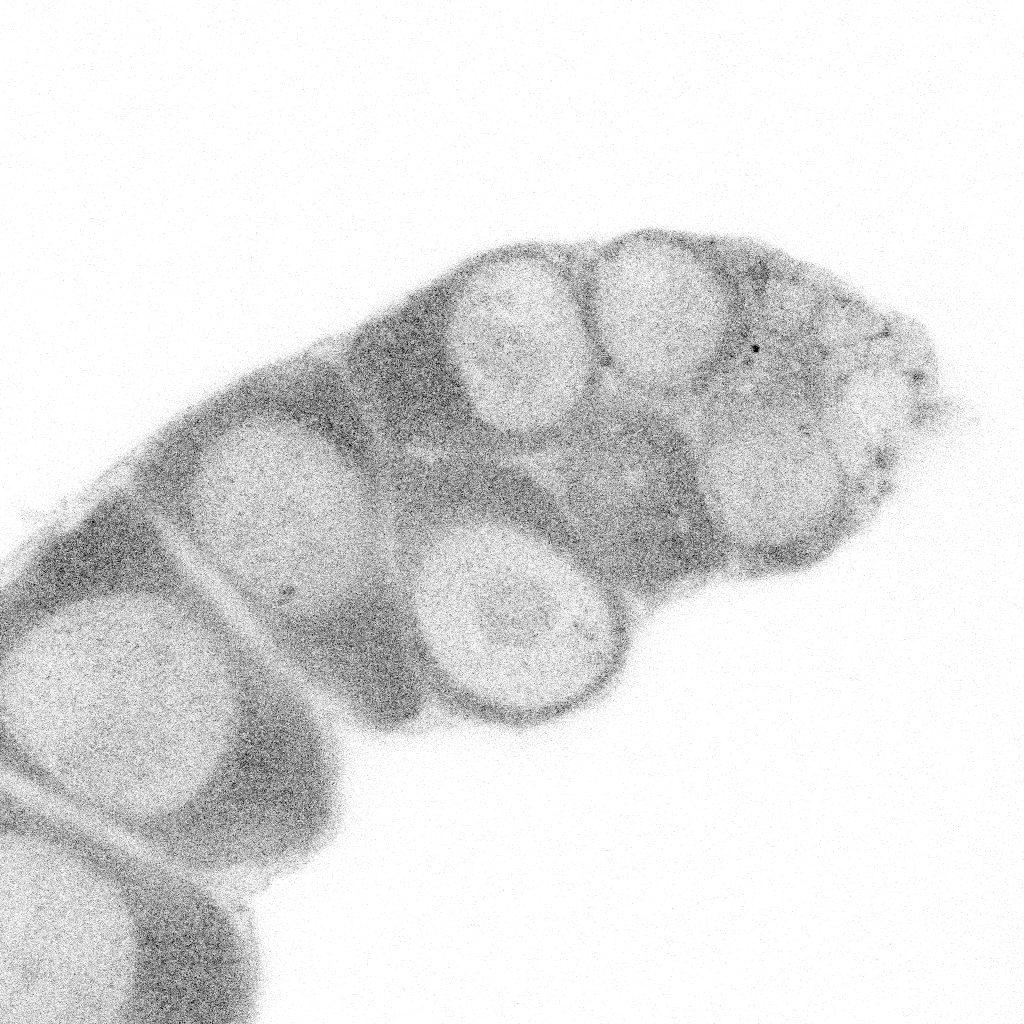

Supplement: Supplementary file 9 — Figure EV3 Source Data [file 44319_2026_741_MOESM9_ESM.zip › Figure_EV3/EV3F/Acheta_domesticus_control_RNA-FISH_FISH-only.tif]

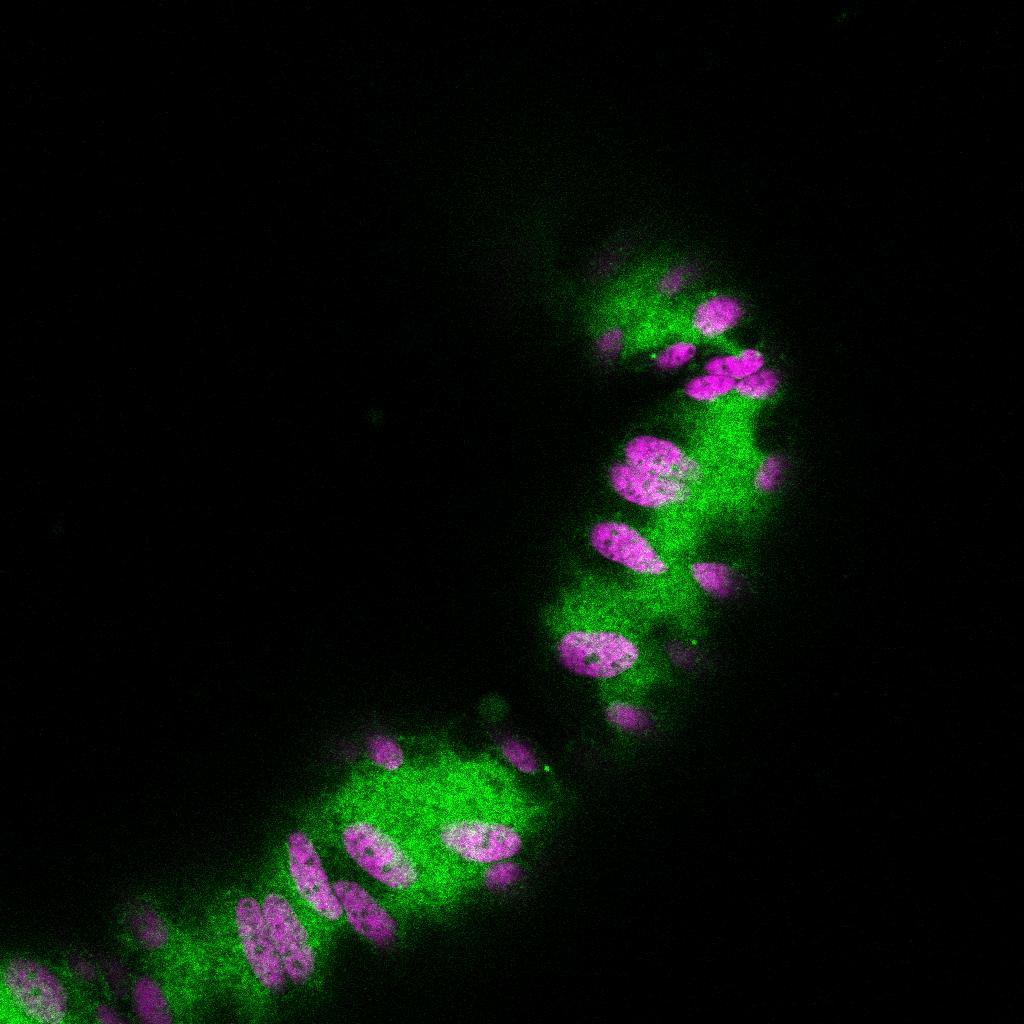

Supplement: Supplementary file 9 — Figure EV3 Source Data [file 44319_2026_741_MOESM9_ESM.zip › Figure_EV3/EV3F/Acheta_domesticus_control_RNA-FISH_birds-eye_FISH-DAPI.tif]

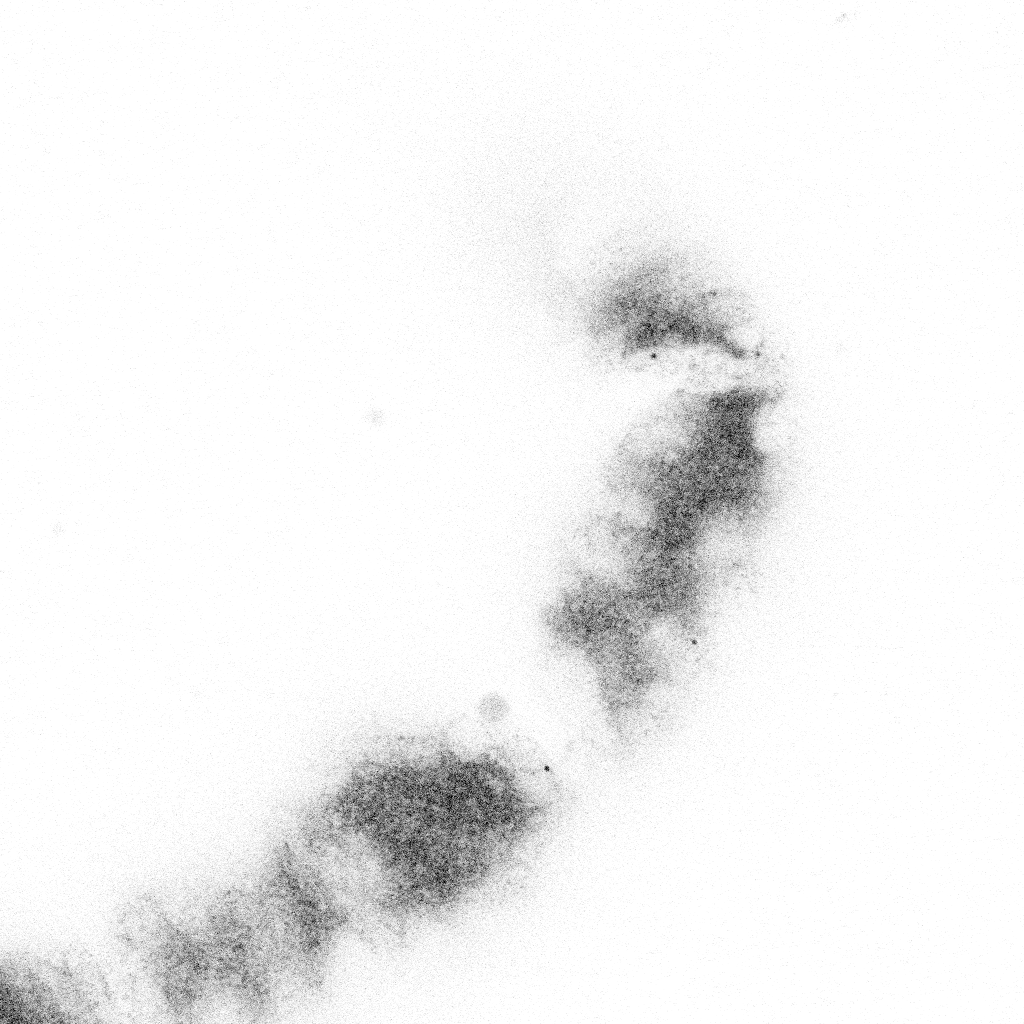

Supplement: Supplementary file 9 — Figure EV3 Source Data [file 44319_2026_741_MOESM9_ESM.zip › Figure_EV3/EV3F/Acheta_domesticus_control_RNA-FISH_birds-eye_FISH-only.tif]

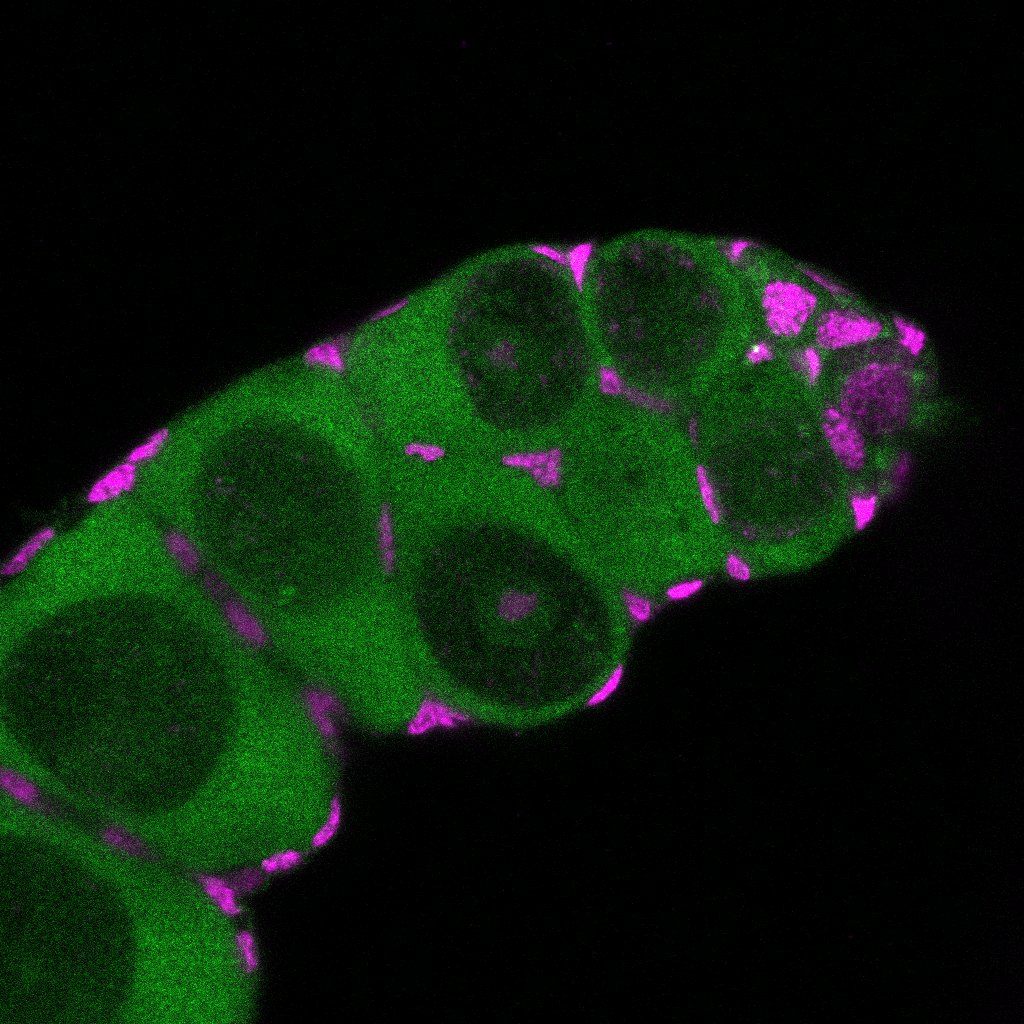

Supplement: Supplementary file 9 — Figure EV3 Source Data [file 44319_2026_741_MOESM9_ESM.zip › Figure_EV3/EV3F/Acheta_domesticus_control_RNA-FISH_FISH-DAPI.tif]

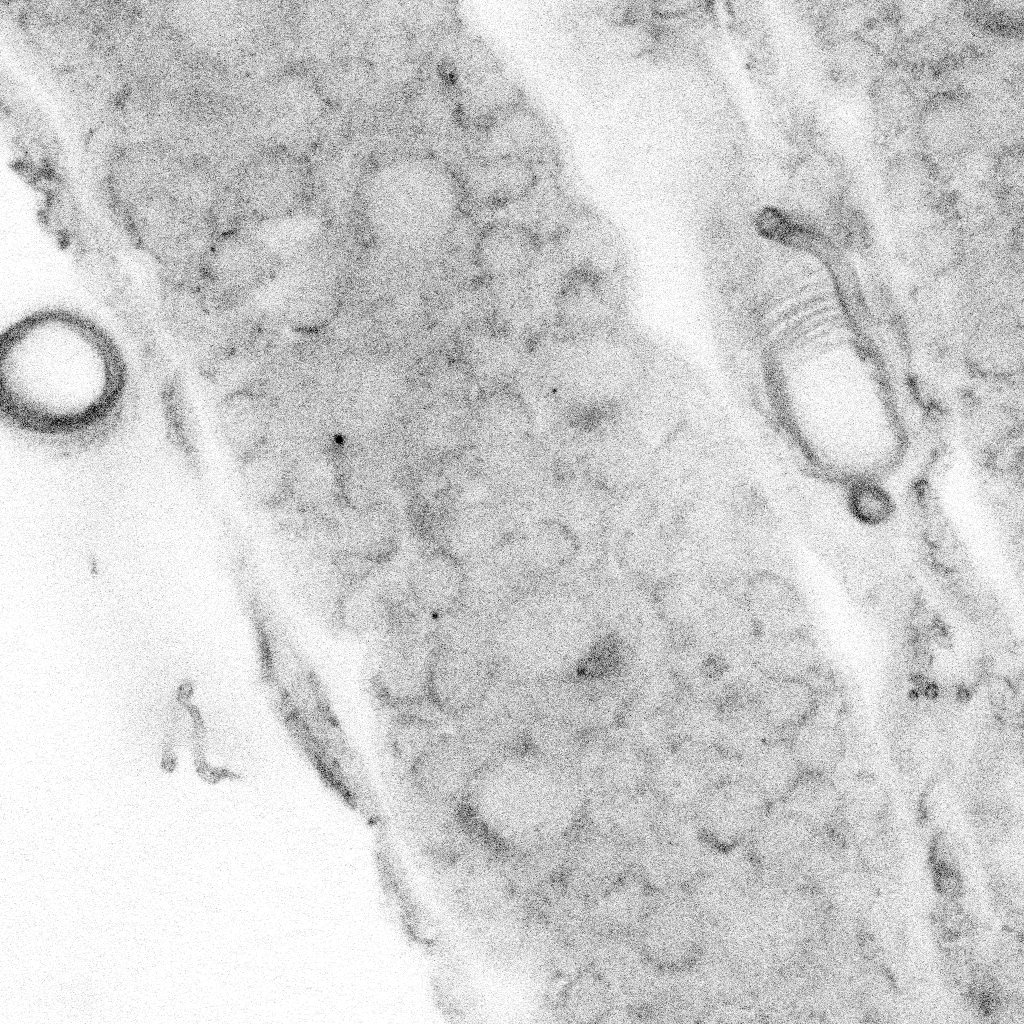

Supplement: Supplementary file 9 — Figure EV3 Source Data [file 44319_2026_741_MOESM9_ESM.zip › Figure_EV3/EV3B/Tetragonula_carbonaria_control_RNA-FISH_FISH-only.tif]

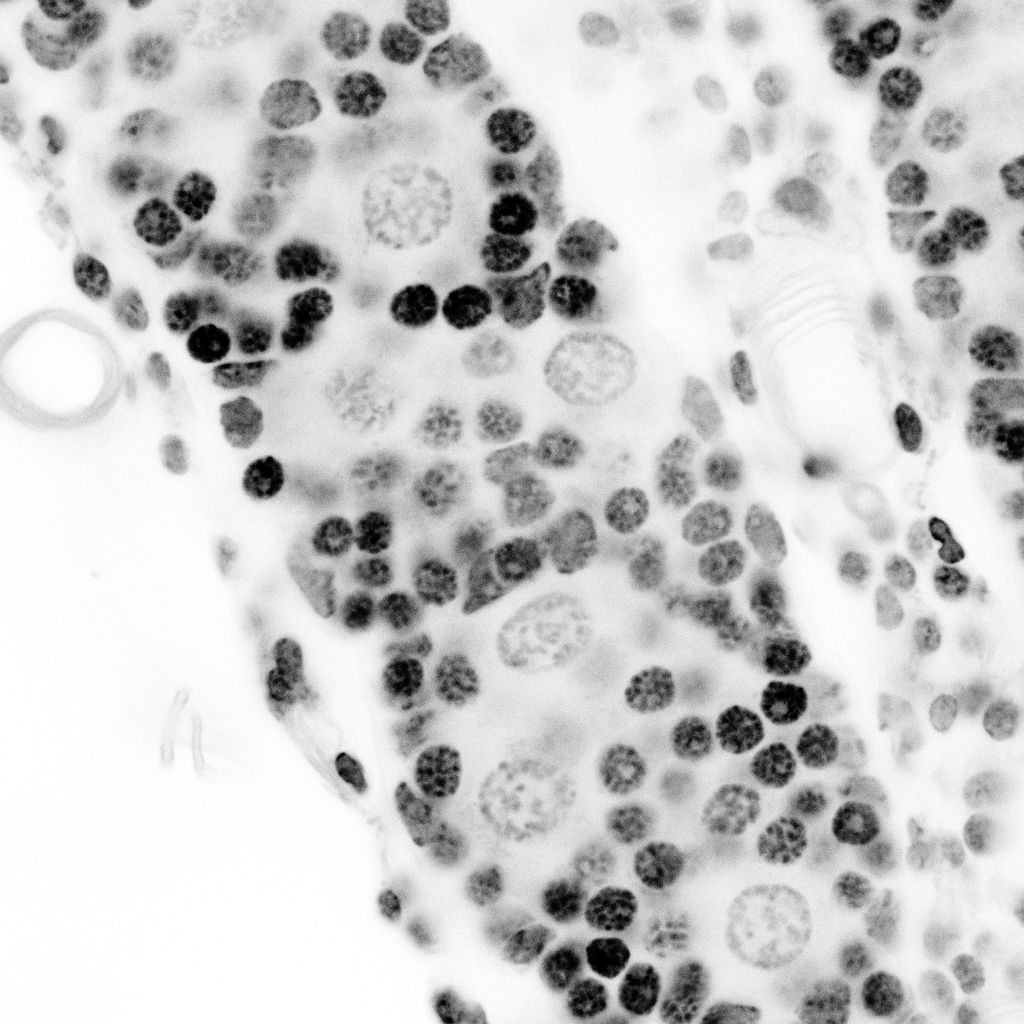

Supplement: Supplementary file 9 — Figure EV3 Source Data [file 44319_2026_741_MOESM9_ESM.zip › Figure_EV3/EV3B/Tetragonula_carbonaria_control_RNA-FISH_DAPI-only.tif]

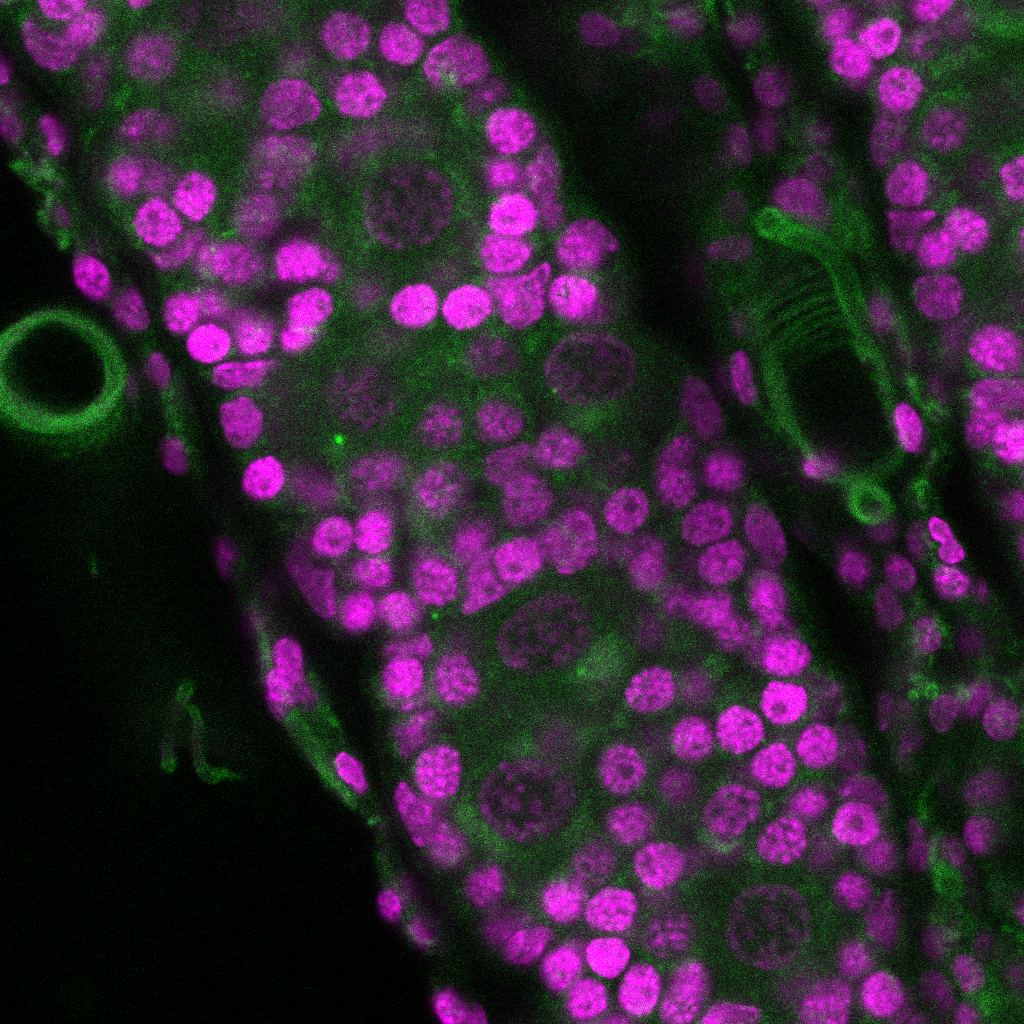

Supplement: Supplementary file 9 — Figure EV3 Source Data [file 44319_2026_741_MOESM9_ESM.zip › Figure_EV3/EV3B/Tetragonula_carbonaria_control_RNA-FISH_FISH-DAPI.tif]

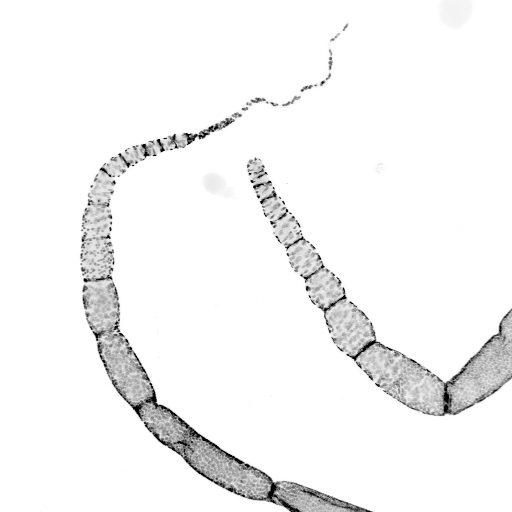

Supplement: Supplementary file 9 — Figure EV3 Source Data [file 44319_2026_741_MOESM9_ESM.zip › Figure_EV3/EV3E/Acheta_domesticus_whole-ovary_DAPI.tif]

Figure EV3E

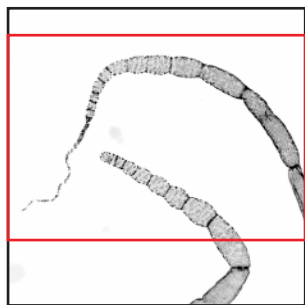

Supplement: Supplementary file 9 — Figure EV3 Source Data [file 44319_2026_741_MOESM9_ESM.zip › Figure_EV3/EV3E/microscopy-images_cropping-information_FigEV3E.pdf]
