# Supplementary material for: Conserved but mechanistically diverse piRNA defence against endogenous retroviruses in insects
Source: EMBO Rep. 2026 Apr 2;27(9):2243–73. doi: 10.1038/s44319-026-00741-4 (PMC13172572; doi:10.1038/s44319-026-00741-4)
Supplement: Supplementary file 10 — Expanded View Figures [file 44319_2026_741_MOESM10_ESM.pdf]

Expanded View Figures

Figure EV1. Putative somatic piRNA clusters of *Eristalis tenax* and *Bactrocera tryoni.*

(**A**) A scatter plot of the second replicate of ovarian and embryonic small RNA libraries of *E. tenax* showing the abundance of piRNAs from the ovaries (X axis) and the embryos (Y axis) that uniquely mapped to the individual 0.5 kb tiles. Tiles from newly identified clusters are highlighted in different colours. (**B**, **C**) piRNA coverage plots across the two other putative somatic piRNA clusters of *E. tenax*—HG993127.1 left in (**B**) and HG993129.1 in (**C**)—shown in counts per million genome mappers (cpm) from replicate 1 of the ovarian small RNA libraries. Reads that mapped to the plus- and minus-strands are coloured in dark and light grey, respectively. (**D**, **D′**) Scatter plots of the first and second replicates of ovarian and embryonic small RNA libraries of *B. tryoni* comparing the abundance of piRNAs from the ovaries (X axis) and the embryos (Y axis) that uniquely mapped to the individual 0.5 kb tiles. Tiles from newly identified clusters are highlighted in different colours. (**E**, **F**) piRNA coverage plots across the two putative somatic piRNA clusters of *B. tryoni*—NC_052502.1 in (**B**) and NC_052503.1 in (**C**)—shown in counts per million genome mappers (cpm) from replicate 1 of the ovarian small RNA libraries. Reads that mapped to the plus- and minus-strands are coloured in dark and light grey, respectively. For (**B**), (**C**), (**E**) and (**F**), coloured bars indicate *gypsy* insertions predicted by RepeatMasker and *gypsy* GAG, POL and ENV open reading frames predicted by tBLASTn.

© The Author(s)

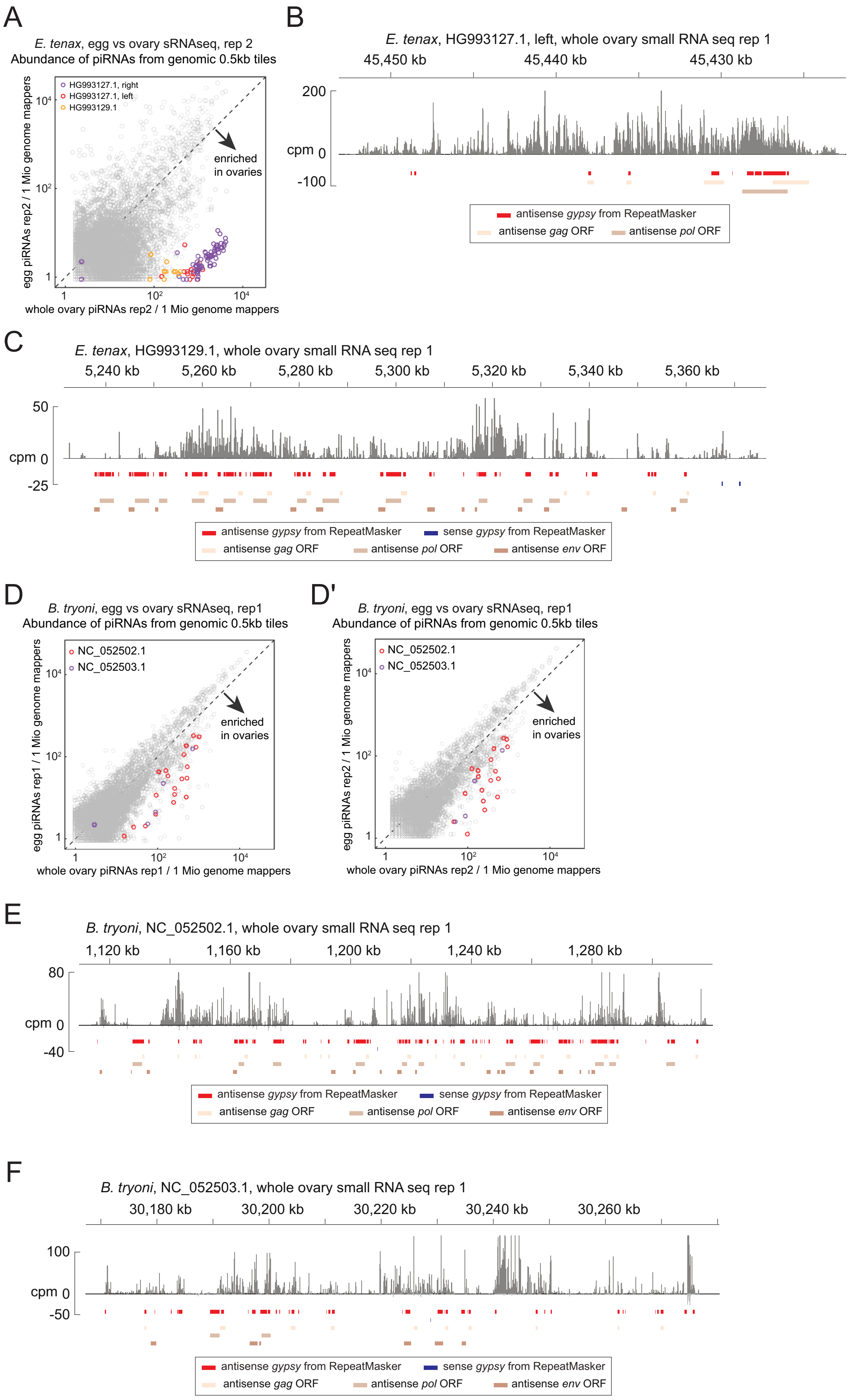
A
E. tenax, egg vs ovary sRNAseq, rep 2
Abundance of piRNAs from genomic 0.5kb tiles
HG993127.1, right
HG993127.1, left
HG993129.1
enriched in ovaries
egg piRNAs rep2 / 1 Mio genome mappers
whole ovary piRNAs rep2 / 1 Mio genome mappers
B
E. tenax, HG993127.1, left, whole ovary small RNA seq rep 1
45,450 kb
45,440 kb
45,430 kb
200
cpm 0
-100
antisense gypsy from RepeatMasker
antisense gag ORF
antisense pol ORF
C
E. tenax, HG993129.1, whole ovary small RNA seq rep 1
5,240 kb
5,260 kb
5,280 kb
5,300 kb
5,320 kb
5,340 kb
5,360 kb
50
cpm 0
-25
antisense gypsy from RepeatMasker
sense gypsy from RepeatMasker
antisense gag ORF
antisense pol ORF
antisense env ORF
D
B. tryoni, egg vs ovary sRNAseq, rep1
Abundance of piRNAs from genomic 0.5kb tiles
NC_052502.1
NC_052503.1
enriched in ovaries
egg piRNAs rep1 / 1 Mio genome mappers
whole ovary piRNAs rep1 / 1 Mio genome mappers
D'
B. tryoni, egg vs ovary sRNAseq, rep1
Abundance of piRNAs from genomic 0.5kb tiles
NC_052502.1
NC_052503.1
enriched in ovaries
egg piRNAs rep2 / 1 Mio genome mappers
whole ovary piRNAs rep2 / 1 Mio genome mappers
E
B. tryoni, NC_052502.1, whole ovary small RNA seq rep 1
1,120 kb
1,160 kb
1,200 kb
1,240 kb
1,280 kb
80
cpm 0
-40
antisense gypsy from RepeatMasker
sense gypsy from RepeatMasker
antisense gag ORF
antisense pol ORF
antisense env ORF
F
B. tryoni, NC_052503.1, whole ovary small RNA seq rep 1
30,180 kb
30,200 kb
30,220 kb
30,240 kb
30,260 kb
100
cpm 0
-50
antisense gypsy from RepeatMasker
sense gypsy from RepeatMasker
antisense gag ORF
antisense pol ORF
antisense env ORF

© The Author(s)

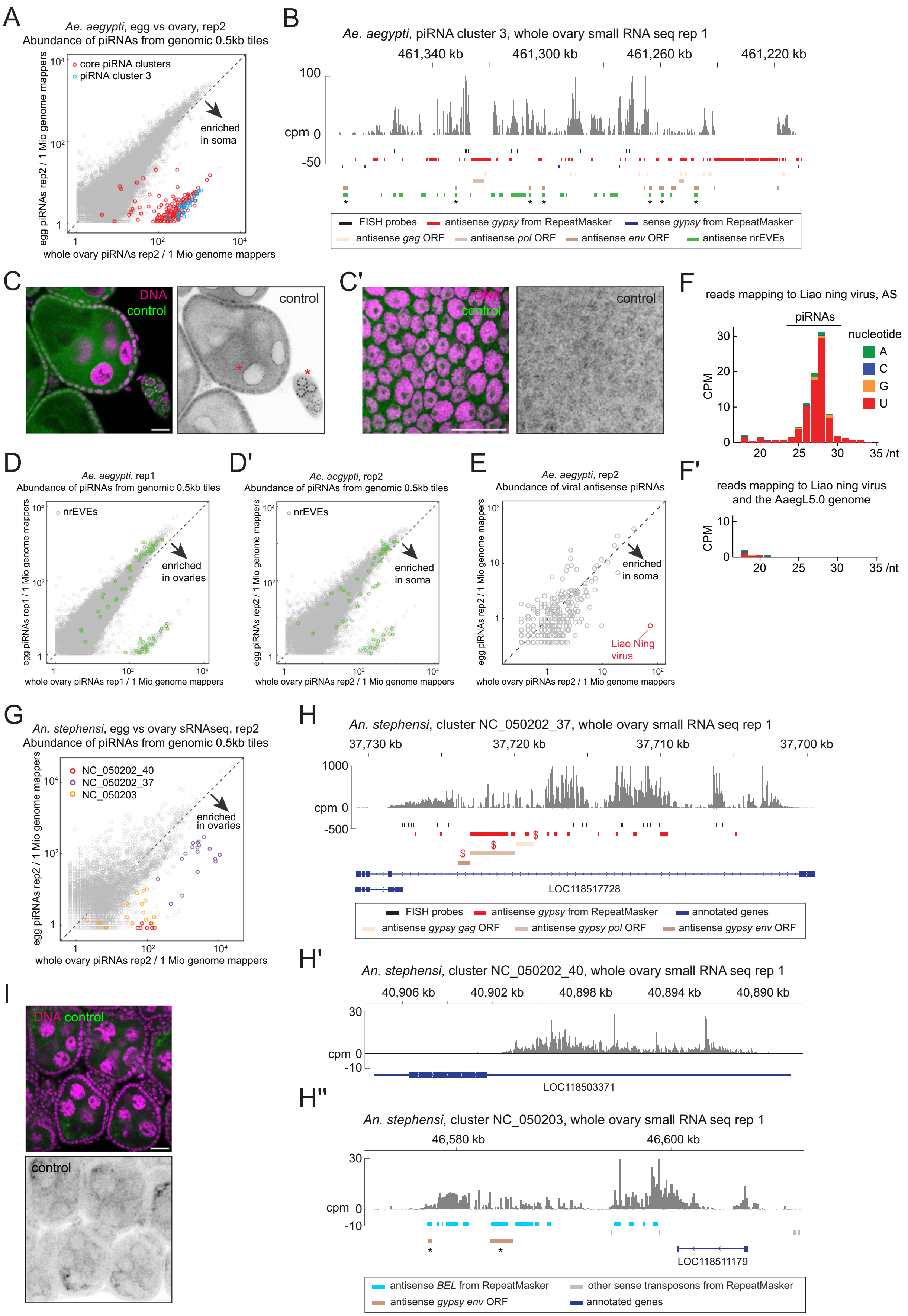
A
Ae. aegypti, egg vs ovary, rep2
Abundance of piRNAs from genomic 0.5kb tiles
core piRNA clusters
piRNA cluster 3
enriched in soma
egg piRNAs rep2 / 1 Mio genome mappers
whole ovary piRNAs rep2 / 1 Mio genome mappers
B
Ae. aegypti, piRNA cluster 3, whole ovary small RNA seq rep 1
461,340 kb
461,300 kb
461,260 kb
461,220 kb
100
cpm 0
-50
FISH probes
antisense gypsy from RepeatMasker
sense gypsy from RepeatMasker
antisense gag ORF
antisense pol ORF
antisense env ORF
antisense nrEVEs
C
DNA
control
control
C'
DNA
control
control
F
reads mapping to Liao ning virus, AS
piRNAs
nucleotide
A
C
G
U
CPM
35 /nt
F'
reads mapping to Liao ning virus and the AaegL5.0 genome
CPM
35 /nt
D
Ae. aegypti, rep1
Abundance of piRNAs from genomic 0.5kb tiles
nrEVEs
enriched in ovaries
egg piRNAs rep1 / 1 Mio genome mappers
whole ovary piRNAs rep1 / 1 Mio genome mappers
D'
Ae. aegypti, rep2
Abundance of piRNAs from genomic 0.5kb tiles
nrEVEs
enriched in soma
egg piRNAs rep2 / 1 Mio genome mappers
whole ovary piRNAs rep2 / 1 Mio genome mappers
E
Ae. aegypti, rep2
Abundance of viral antisense piRNAs
enriched in soma
Liao Ning virus
egg piRNAs rep2 / 1 Mio genome mappers
whole ovary piRNAs rep2 / 1 Mio genome mappers
G
An. stephensi, egg vs ovary sRNAseq, rep2
Abundance of piRNAs from genomic 0.5kb tiles
NC_050202_40
NC_050202_37
NC_050203
enriched in ovaries
egg piRNAs rep2 / 1 Mio genome mappers
whole ovary piRNAs rep2 / 1 Mio genome mappers
H
An. stephensi, cluster NC_050202_37, whole ovary small RNA seq rep 1
37,730 kb
37,720 kb
37,710 kb
37,700 kb
1000
cpm 0
-500
LOC118517728
FISH probes
antisense gypsy from RepeatMasker
annotated genes
antisense gypsy gag ORF
antisense gypsy pol ORF
antisense gypsy env ORF
H'
An. stephensi, cluster NC_050202_40, whole ovary small RNA seq rep 1
40,906 kb
40,902 kb
40,898 kb
40,894 kb
40,890 kb
30
cpm 0
-10
LOC118503371
H''
An. stephensi, cluster NC_050203, whole ovary small RNA seq rep 1
46,580 kb
46,600 kb
30
cpm 0
-10
LOC118511179
antisense BEL from RepeatMasker
other sense transposons from RepeatMasker
antisense gypsy env ORF
annotated genes
I
DNA control
control

Figure EV2. Characterisation of the ovarian somatic piRNA clusters of *Aedes aegypti* and *Anopheles stephensi*.

(**A**) Scatter plots for the replicate 2 of the small RNA sequencing of *Ae. aegypti* showing the ovarian somatic piRNA cluster tiles, equivalent to Fig. 2A. (**B**) Shown is the coverage of piRNA reads (> 22nt) in counts per million genome mappers (cpm) from the ovarian small RNA library of *Ae. aegypti* that uniquely mapped to the piRNA cluster 3. Reads that mapped to the plus- and minus-strands are coloured in dark and light grey, respectively. Coloured bars indicate *gypsy* insertions predicted by RepeatMasker, *gypsy* GAG, POL and ENV open reading frames predicted by tBLASTn, non-retroviral endogenous viral elements (nrEVEs) from Russo et al (2019) and the FISH probes. Some regions are predicted for *gypsy* elements and RNA viruses because they both have type III fusion glycoproteins (indicated by asterisks). (**C**, **C′**) Side (**C**) and bird's-eye (**C′**) views of RNA FISH images of *Ae. aegypti* egg chambers using probes against *A. domesticus piwi1* mRNA as a negative control. No specific signals are observed except for the staining around the germline nuclei (asterisks). Scale bars = 20 µm. (**D**, **D′**) Scatter plots as in (A and Fig. 2A) but highlighting tiles in which more than half of the regions are occupied by nrEVEs. (**E**) Scatter plot for the replicate 2 showing the abundance of piRNAs mapping to viruses, equivalent to Fig. 2D. (**F**, **F′**) Shown are the size distribution and 5′-end nucleotide frequency of small RNA reads mapping to the antisense strand of Liao ning virus segment 5 in (**F**), and of those additionally mapping to the *Ae. aegypti* genome in (**F′**). (**G**) A scatter plot for the replicate 2 of the small RNA sequencing of *An. stephensi*, equivalent to Fig. 2G, showing the ovary-enriched somatic piRNA cluster tiles. (**H**–**H″**) piRNA coverage plots across three putative somatic piRNA clusters of *An. stephensi*—NC_050202_37 in (**H**), NC_050202_40 in (**H′**), and NC_050203 in (**H″**)—shown in counts per million genome mappers (cpm) from replicate 1 of the ovarian small RNA library. Reads that mapped to the plus- and minus-strands are coloured in dark and light grey, respectively. Coloured bars indicate *gypsy* and *BEL* insertions predicted by RepeatMasker and *gypsy* GAG, POL and ENV open reading frames predicted by tBLASTn, annotated genes and the FISH probes. Some regions are predicted for *gypsy* and *BEL* because they both have type III fusion glycoproteins (indicated by asterisks). Antisense *gypsy* fragments that were examined for corresponding full-length insertions in Fig. EV5 are marked by dollars. (**I**) A control RNA FISH image of *An. stephensi* egg chambers using probes against *A. domesticus piwi1* mRNA. Scale bar = 20 µm. Source data are available online for this figure.

 © The Author(s)

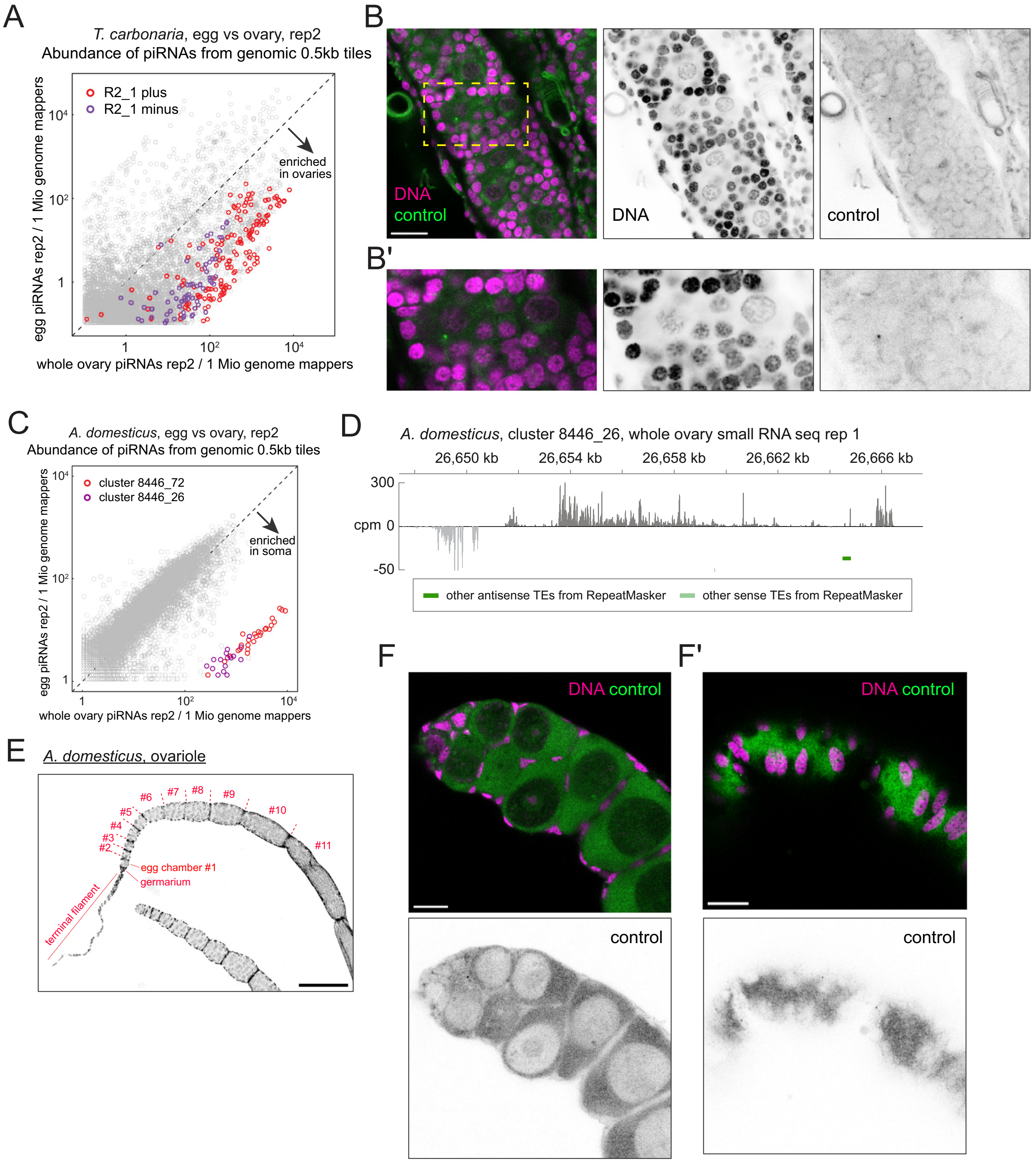
A
T. carbonaria, egg vs ovary, rep2
Abundance of piRNAs from genomic 0.5kb tiles
R2_1 plus
R2_1 minus
enriched in ovaries
egg piRNAs rep2 / 1 Mio genome mappers
whole ovary piRNAs rep2 / 1 Mio genome mappers
B
DNA
control
B'
C
A. domesticus, egg vs ovary, rep2
Abundance of piRNAs from genomic 0.5kb tiles
cluster 8446_72
cluster 8446_26
enriched in soma
D
A. domesticus, cluster 8446_26, whole ovary small RNA seq rep 1
26,650 kb
26,654 kb
26,658 kb
26,662 kb
26,666 kb
cpm
other antisense TEs from RepeatMasker
other sense TEs from RepeatMasker
E
A. domesticus, ovariole
egg chamber #1
germarium
terminal filament
F
F'
DNA control
control

Figure EV3. Characterisation of the ovarian somatic piRNA clusters of stingless bees and crickets.

(**A**) A scatter plot for the replicate 2 of the small RNA sequencing of *T. carbonaria*, equivalent to Fig. 3A, showing the tiles from the ovary-enriched somatic piRNA cluster R2_1. (**B**) A control RNA FISH image of *T. carbonaria* egg chambers using probes against *A. domesticus piwi1* mRNA. (**C**) A scatter plot for the replicate 2 of the small RNA sequencing of *A. domesticus*, equivalent to Fig. 4A, showing the ovary-enriched somatic piRNA cluster tiles. (**D**) A piRNA coverage plot across the somatic piRNA cluster 8446_26 of *A. domesticus* shown in counts per million genome mappers (cpm) from replicate 1 of the ovarian small RNA library. Reads that mapped to the plus- and minus-strands are coloured in dark and light grey, respectively. (**E**) A confocal image showing an ovariole of *A. domesticus* stained by DAPI. The terminal filament, germarium and developing egg chambers are indicated. (**F**, **F′**) Control RNA FISH images of *A. domesticus* egg chambers using probes against *D. melanogaster piwi* mRNA. Scale bars = 20 µm. Source data are available online for this figure.

 © The Author(s)

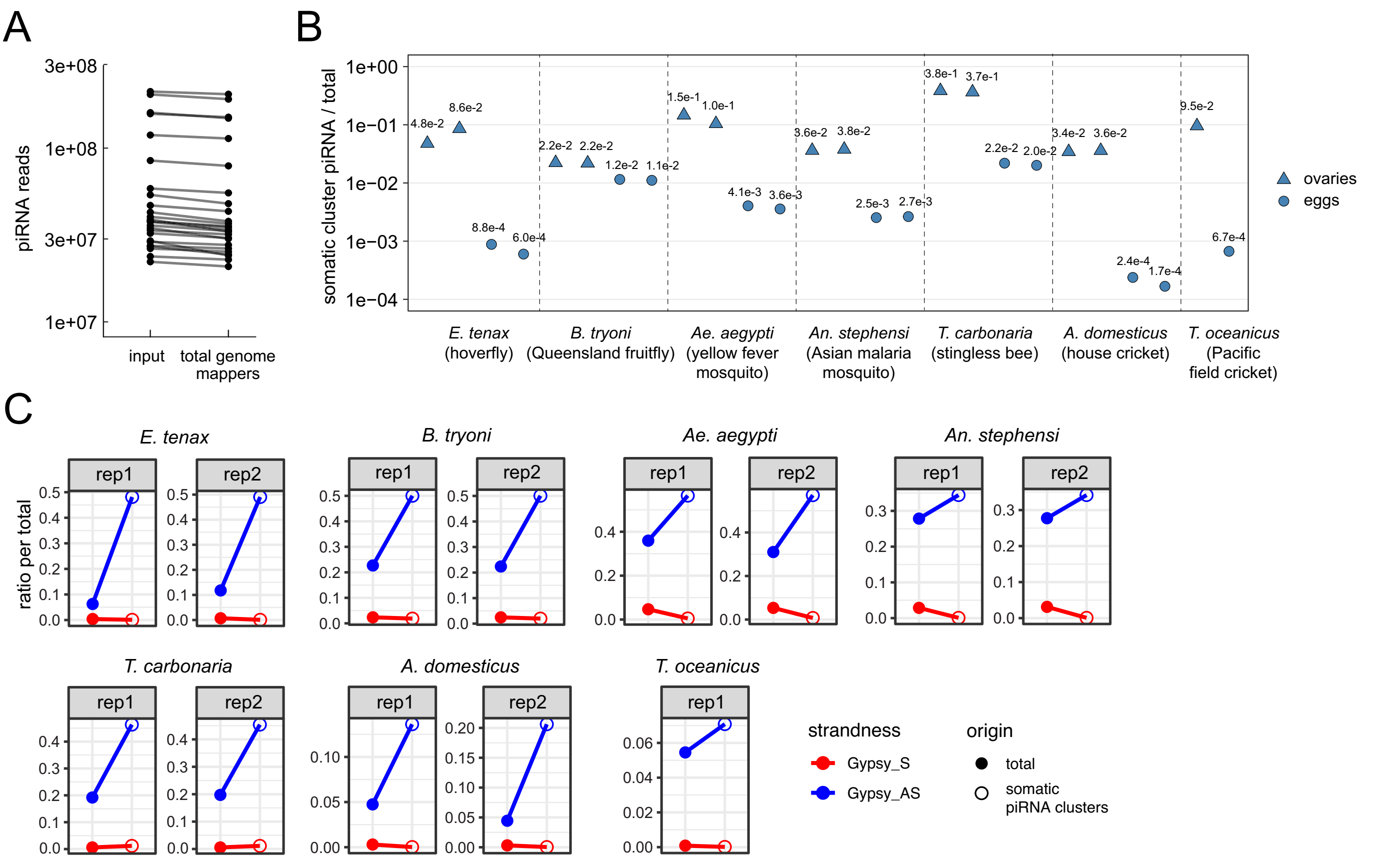

Figure EV4. Ovarian somatic cells selectively express antisense *gypsy* piRNAs across insects.

(**A**) Shown are the number of reads (> 22nt) sequenced (input) and mapped to the respective genomes of all 26 small RNA sequencing libraries analysed in this study. (**B**) Shown are the fraction of piRNA reads (> 22nt) from ovarian and embryonic small RNA sequencing mapping to the ovarian somatic piRNA clusters of the respective genomes out of all genome mapping piRNAs. (**C**) Shown are the fraction of piRNA reads originating from sense and antisense strands of *gypsy* insertions out of all genome-mapping piRNAs. The coverage of reads mapping to multiple genomic loci was normalised by the number of mapping instances per read for (**B**) and (**C**).

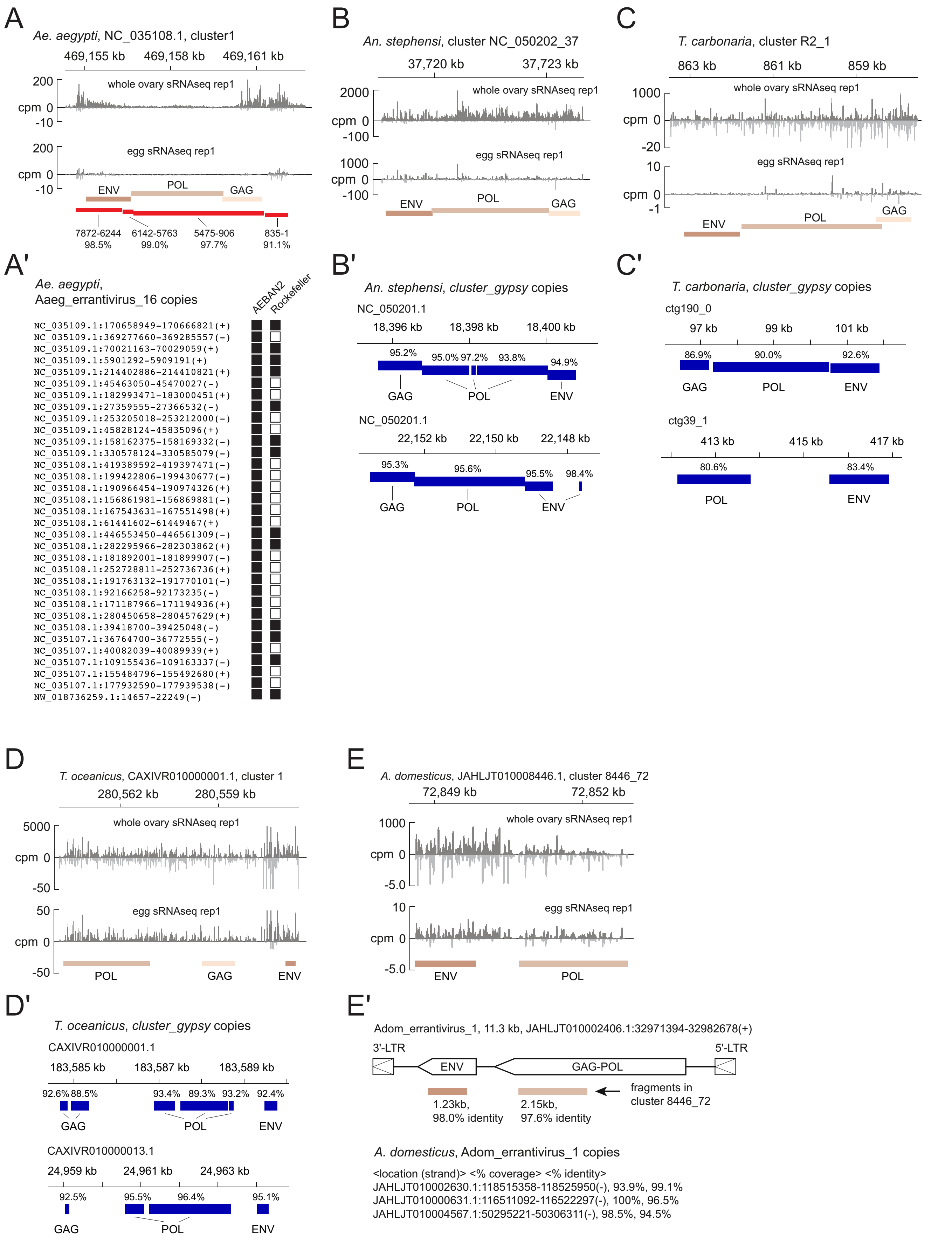
A
Ae. aegypti, NC_035108.1, cluster1
469,155 kb 469,158 kb 469,161 kb
200
cpm 0
-10
whole ovary sRNAseq rep1
200
cpm 0
-10
egg sRNAseq rep1
ENV
POL
GAG
7872-6244 98.5%
6142-5763 99.0%
5475-906 97.7%
835-1 91.1%
B
An. stephensi, cluster NC_050202_37
37,720 kb 37,723 kb
2000
cpm 0
-100
whole ovary sRNAseq rep1
1000
cpm 0
-100
egg sRNAseq rep1
ENV
POL
GAG
C
T. carbonaria, cluster R2_1
863 kb 861 kb 859 kb
1000
cpm 0
-20
whole ovary sRNAseq rep1
10
cpm 0
-1
egg sRNAseq rep1
ENV
POL
GAG
A'
Ae. aegypti,
Aaeg_errantivirus_16 copies
AEBAN2
Rockefeller
NC_035109.1:170658949-170666821(+)
NC_035109.1:369277660-369285557(-)
NC_035109.1:70021163-70029059(+)
NC_035109.1:5901292-5909191(+)
NC_035109.1:214402886-214410821(+)
NC_035109.1:45463050-45470027(-)
NC_035109.1:182993471-183000451(+)
NC_035109.1:27359555-27366532(-)
NC_035109.1:253205018-253212000(-)
NC_035109.1:45828124-45835096(+)
NC_035109.1:158162375-158169332(-)
NC_035109.1:330578124-330585079(-)
NC_035108.1:419389592-419397471(-)
NC_035108.1:199422806-199430677(-)
NC_035108.1:190966454-190974326(+)
NC_035108.1:156861981-156869881(-)
NC_035108.1:167543631-167551498(+)
NC_035108.1:61441602-61449467(+)
NC_035108.1:446553450-446561309(-)
NC_035108.1:282295966-282303862(+)
NC_035108.1:181892001-181899907(-)
NC_035108.1:252728811-252736736(+)
NC_035108.1:191763132-191770101(-)
NC_035108.1:92166258-92173235(-)
NC_035108.1:171187966-171194936(+)
NC_035108.1:280450658-280457629(+)
NC_035108.1:39418700-39425048(-)
NC_035107.1:36764700-36772555(-)
NC_035107.1:40082039-40089939(+)
NC_035107.1:109155436-109163337(-)
NC_035107.1:155484796-155492680(+)
NC_035107.1:177932590-177939538(-)
NW_018736259.1:14657-22249(-)
B'
An. stephensi, cluster_gypsy copies
NC_050201.1
18,396 kb 18,398 kb 18,400 kb
95.2% 95.0% 97.2% 93.8% 94.9%
GAG POL ENV
NC_050201.1
22,152 kb 22,150 kb 22,148 kb
95.3% 95.6% 95.5% 98.4%
GAG POL ENV
C'
T. carbonaria, cluster_gypsy copies
ctg190_0
97 kb 99 kb 101 kb
86.9% 90.0% 92.6%
GAG POL ENV
ctg39_1
413 kb 415 kb 417 kb
80.6% 83.4%
POL ENV
D
T. oceanicus, CAXIVR010000001.1, cluster 1
280,562 kb 280,559 kb
5000
cpm 0
-50
whole ovary sRNAseq rep1
50
cpm 0
-50
egg sRNAseq rep1
POL GAG ENV
E
A. domesticus, JAHLJT010008446.1, cluster 8446_72
72,849 kb 72,852 kb
1000
cpm 0
-5.0
whole ovary sRNAseq rep1
10
cpm 0
-5.0
egg sRNAseq rep1
ENV POL
D'
T. oceanicus, cluster_gypsy copies
CAXIVR010000001.1
183,585 kb 183,587 kb 183,589 kb
92.6% 88.5% 93.4% 89.3% 93.2% 92.4%
GAG POL ENV
CAXIVR010000013.1
24,959 kb 24,961 kb 24,963 kb
92.5% 95.5% 96.4% 95.1%
GAG POL ENV
E'
Adom_errantivirus_1, 11.3 kb, JAHLJT010002406.1:32971394-32982678(+)
3'-LTR
5'-LTR
ENV
GAG-POL
1.23kb, 98.0% identity
2.15kb, 97.6% identity
fragments in cluster 8446_72
A. domesticus, Adom_errantivirus_1 copies
<location (strand)> <% coverage> <% identity>
JAHLJT010002630.1:118515358-118525950(-), 93.9%, 99.1%
JAHLJT010000631.1:116511092-116522297(-), 100%, 96.5%
JAHLJT010004567.1:50295221-50306311(-), 98.5%, 94.5%

© The Author(s)

Figure EV5. Genomic *gypsy* insertions that are targeted by ovarian somatic piRNAs.

(**A**) Shown are *gypsy* GAG, POL and ENV insertions in cluster 1 of the *Ae. aegypti* genome that are homologous to the previously identified *env*-containing *gypsy* element *Aaeg_errantivirus_16* (Chary and Hayashi, 2025). Nucleotide positions and percent sequence identities of the homologous regions are shown. The piRNA read coverage from ovarian and embryonic small RNA libraries is shown. The coverage of reads mapping to multiple genomic loci was normalised by the number of mapping instances per read across all panels in this figure. (**A′**) Shown are the genomic positions of 33 *Aaeg_errantivirus_16* copies in the AaegL5.0 genome assembly (Liverpool strain). Presence and absence of the syntenic copies in the AEBAN2 isolate (IBAB_Aaeg_KPA_1.0) and the Rockefeller strain (CU_AaegROCK_1.0) are shown in filled and open boxes, respectively. (**B–E**) Shown are the piRNA read coverage of the ovarian and embryonic small RNA sequencing libraries from *An. stephensi* (**B**), *T. carbonaria* (**C**), *T. oceanicus* (**D**) and *A. domesticus* (**E**), across the *gypsy* GAG, POL and ENV insertions that are tandemly aligned in ovarian somatic piRNA clusters. Positions of these insertions in the cluster are shown in Fig. EV2 for *An. stephensi*, Fig. 3 for *T. carbonaria* and Fig. 4 for *T. oceanicus* and *A. domesticus*. (**B′–E′**) Shown are *gypsy* copies of the respective genomes harbouring GAG, POL and ENV open reading frames that are each homologous to the insertions found in the piRNA clusters. Sequence identifies to the cluster insertions are shown in percentage. Cluster insertions of ENV and POL in *A. domesticus* are homologous to an *env*-containing *gypsy* element, which we named as *Adom_errantivirus_1*. The *A. domesticus* genome carries three copies of *Adom_errantivirus_1* with >90% coverage and >90% sequence identities.

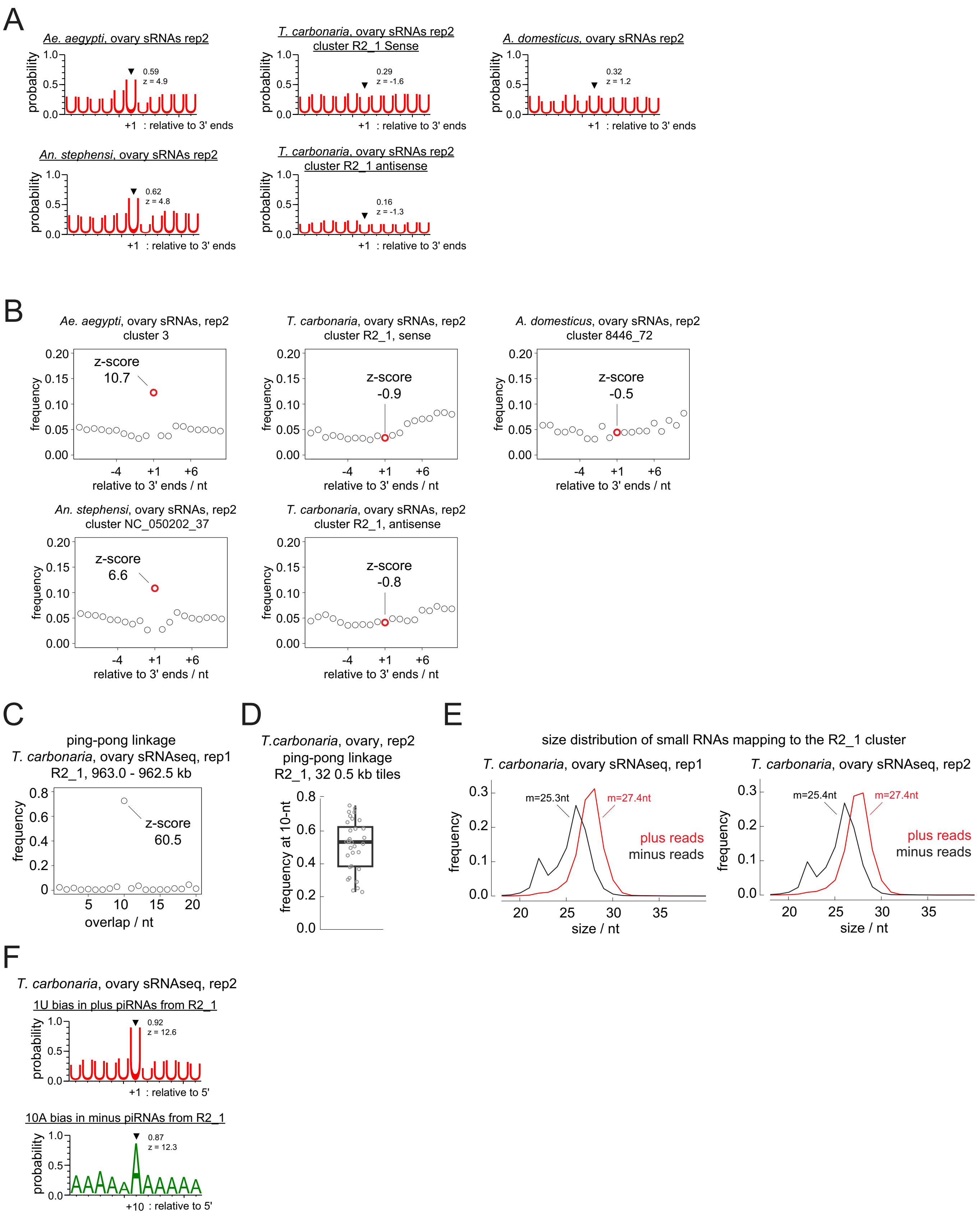
A
Ae. aegypti, ovary sRNAs rep2
probability
1.0
0.5
0.0
0.59
z = 4.9
+1 : relative to 3' ends
T. carbonaria, ovary sRNAs rep2
cluster R2_1 Sense
0.29
z = -1.6
+1 : relative to 3' ends
A. domesticus, ovary sRNAs rep2
0.32
z = 1.2
+1 : relative to 3' ends
An. stephensi, ovary sRNAs rep2
0.62
z = 4.8
+1 : relative to 3' ends
T. carbonaria, ovary sRNAs rep2
cluster R2_1 antisense
0.16
z = -1.3
+1 : relative to 3' ends
B
Ae. aegypti, ovary sRNAs, rep2
cluster 3
frequency
0.20
0.15
0.10
0.05
0.00
z-score
10.7
-4
+1
+6
relative to 3' ends / nt
T. carbonaria, ovary sRNAs, rep2
cluster R2_1, sense
z-score
-0.9
relative to 3' ends / nt
A. domesticus, ovary sRNAs, rep2
cluster 8446_72
z-score
-0.5
relative to 3' ends / nt
An. stephensi, ovary sRNAs, rep2
cluster NC_050202_37
z-score
6.6
relative to 3' ends / nt
T. carbonaria, ovary sRNAs, rep2
cluster R2_1, antisense
z-score
-0.8
relative to 3' ends / nt
C
ping-pong linkage
T. carbonaria, ovary sRNAseq, rep1
R2_1, 963.0 - 962.5 kb
frequency
0.8
0.6
0.4
0.2
0
z-score
60.5
5
10
15
20
overlap / nt
D
T.carbonaria, ovary, rep2
ping-pong linkage
R2_1, 32 0.5 kb tiles
frequency at 10-nt
0.8
0.6
0.4
0.2
0.0
E
size distribution of small RNAs mapping to the R2_1 cluster
T. carbonaria, ovary sRNAseq, rep1
frequency
0.3
0.2
0.1
0.0
m=25.3nt
m=27.4nt
plus reads
minus reads
20
25
30
35
size / nt
T. carbonaria, ovary sRNAseq, rep2
m=25.4nt
m=27.4nt
plus reads
minus reads
size / nt
F
T. carbonaria, ovary sRNAseq, rep2
1U bias in plus piRNAs from R2_1
probability
1.0
0.5
0.0
0.92
z = 12.6
+1 : relative to 5'
10A bias in minus piRNAs from R2_1
0.87
z = 12.3
+10 : relative to 5'

 © The Author(s)

Figure EV6. Linkage analysis of phasing and ping-pong piRNA biogenesis for ovarian small RNA libraries.

(**A**, **B**) Analyses performed in Fig. 6 were repeated for the second replicates of ovarian small RNA libraries from different insects. Shown in (**A**) are the frequencies of uridines found at positions relative to the 3′ ends of piRNAs mapping to the ovarian somatic piRNA clusters of individual insect species. Frequencies at the linkage position (+1) and the z-scores are shown. In (**B**), frequency plots of the distance between piRNA 3′ and 5′ ends from representative ovarian somatic clusters are shown for individual species. The Z-scores of the linkage distance +1, in which piRNA 5′ ends are found immediately after piRNA 3′ ends, are shown. (**C**) Frequency plot of the 5′–5′ overlaps of piRNAs from the plus- and minus-strands of *T. carbonaria* cluster R2_1, region 963.0 - 962.5 kb. The Z-score of the linkage overlap length—10 nucleotides—is shown. (**D**) Boxplot showing frequencies at the 10-nt overlap from 0.5 kb tiles from cluster R2_1. Circles represent values from individual 0.5 kb genomic tiles from the clusters. The centre line indicates the medians; box limits represent the interquartile range (IQR); whiskers extend to 1.5× IQR. $N = 32$. (**E**) Size distributions of the two replicates of *T. carbonaria* ovarian small RNAs mapping to the plus- and minus-strands of cluster R2_1 are shown with mean lengths. (**F**) Shown are the frequencies of Uridines and Adenosines found at positions around the 5′ end and the tenth nucleotide of piRNAs mapping to the plus- and minus-strands of cluster R2_1. Frequencies at the linkage positions and the z-scores are shown. The analyses performed in Fig. 7C,E were repeated for the second replicate of *T. carbonaria* ovarian small RNA library in (**D**) and (**F**), respectively.

© The Author(s)

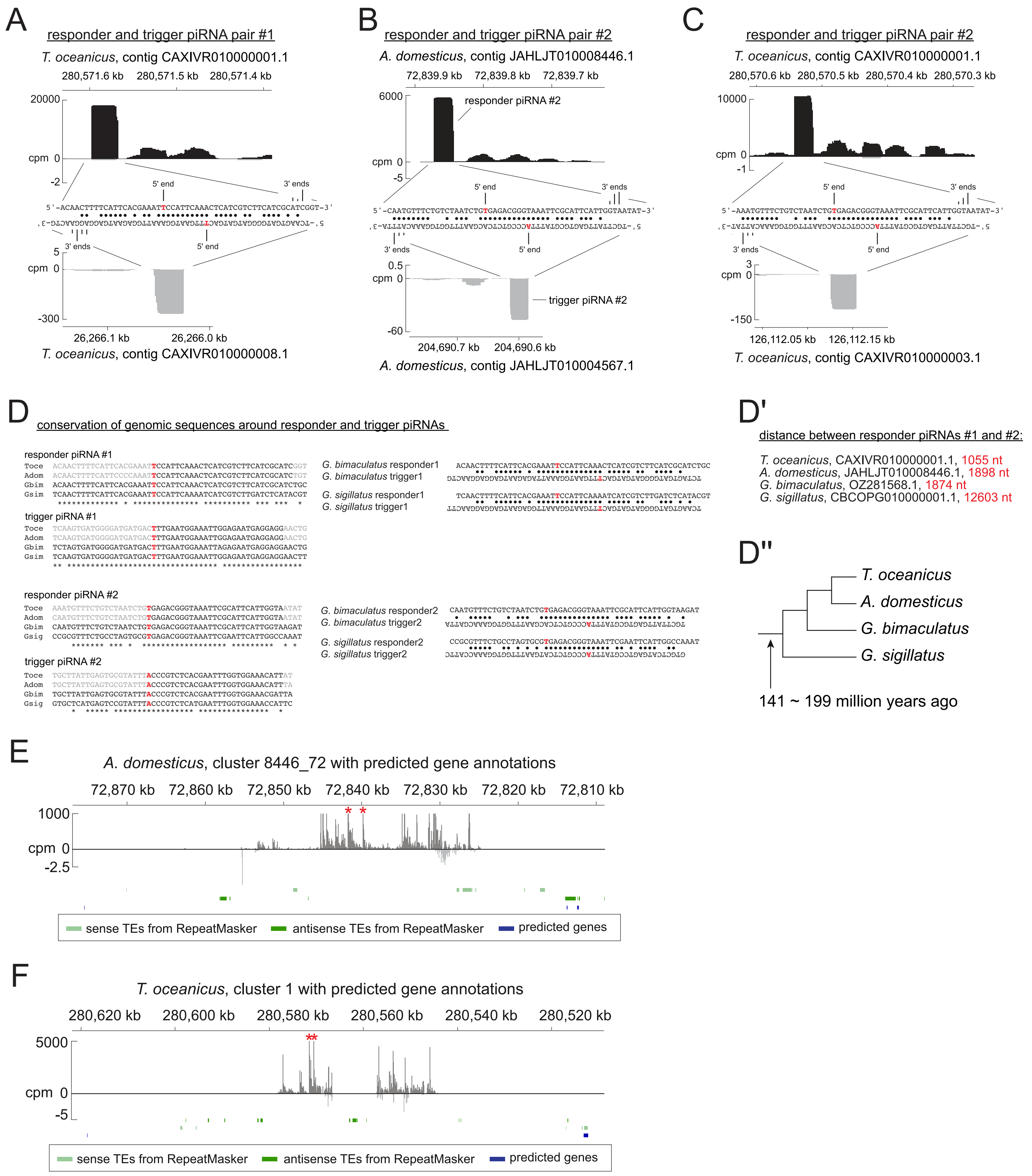
A
responder and trigger piRNA pair #1
T. oceanicus, contig CAXIVR010000001.1
280,571.6 kb 280,571.5 kb 280,571.4 kb
20000
cpm 0
-2
5' end
3' ends
5
cpm 0
-300
26,266.1 kb 26,266.0 kb
T. oceanicus, contig CAXIVR010000008.1
B
responder and trigger piRNA pair #2
A. domesticus, contig JAHLJT010008446.1
72,839.9 kb 72,839.8 kb 72,839.7 kb
6000
responder piRNA #2
cpm 0
-5
0.5
cpm 0
-60
trigger piRNA #2
204,690.7 kb 204,690.6 kb
A. domesticus, contig JAHLJT010004567.1
C
responder and trigger piRNA pair #2
T. oceanicus, contig CAXIVR010000001.1
280,570.6 kb 280,570.5 kb 280,570.4 kb 280,570.3 kb
10000
cpm 0
-1
3
cpm 0
-150
126,112.05 kb 126,112.15 kb
T. oceanicus, contig CAXIVR010000003.1
D
conservation of genomic sequences around responder and trigger piRNAs
responder piRNA #1
trigger piRNA #1
responder piRNA #2
trigger piRNA #2
G. bimaculatus responder1
G. bimaculatus trigger1
G. sigillatus responder1
G. sigillatus trigger1
G. bimaculatus responder2
G. bimaculatus trigger2
G. sigillatus responder2
G. sigillatus trigger2
D'
distance between responder piRNAs #1 and #2:
T. oceanicus, CAXIVR010000001.1, 1055 nt
A. domesticus, JAHLJT010008446.1, 1898 nt
G. bimaculatus, OZ281568.1, 1874 nt
G. sigillatus, CBCOPG010000001.1, 12603 nt
D''
T. oceanicus
A. domesticus
G. bimaculatus
G. sigillatus
141 ~ 199 million years ago
E
A. domesticus, cluster 8446_72 with predicted gene annotations
72,870 kb 72,860 kb 72,850 kb 72,840 kb 72,830 kb 72,820 kb 72,810 kb
1000
cpm 0
-2.5
sense TEs from RepeatMasker
antisense TEs from RepeatMasker
predicted genes
F
T. oceanicus, cluster 1 with predicted gene annotations
280,620 kb 280,600 kb 280,580 kb 280,560 kb 280,540 kb 280,520 kb
5000
cpm 0
-5
sense TEs from RepeatMasker
antisense TEs from RepeatMasker
predicted genes

© The Author(s)

Figure EV7. Conversation of trigger and responder piRNA pairs in cricket ovarian somatic clusters.

(**A–C**) Sequences and the cpm coverage of genomic regions at responder and trigger piRNA pairs #1 from *T. oceanicus* in (**A**), pairs #2 from *A. domesticus* in (**B**) and *T. oceanicus* in (**C**). The 5′ and 3′ end positions are indicated with frequencies represented by heights for the 3′ ends. Watson-Crick base pairs are marked by circles. (**D**) Sequence alignments of the genomic regions at the responder and trigger piRNAs from various cricket species. The two responder piRNAs are derived from the same genomic locus for all four species. (**D′**) Distances between them for each species are shown. (**E**, **F**) The flanking genomic regions of the piRNA clusters possessing the two responder piRNAs (asterisks) are shown for *A. domesticus* in (**E**) and *T. oceanicus* in (**F**) with protein-coding genes predicted by BLASTn search of previously published transcriptome of each species (Bailey et al, 2013; Oppert et al, 2020).
